# Microneedle-mediated transdermal delivery of siRNA-loaded nanoparticles for atopic dermatitis therapy by disrupting cuproptosis-pyroptosis crosstalk

**DOI:** 10.1186/s12951-026-04533-9

**Published:** 2026-05-18

**Authors:** Pian Yu, Chi Fang, Zhisheng Luo, Lu Hao, Kaixuan Li, Rongxuan Yan, Sihui Ma, Guanming Wang, Qiaozhi Cao, Jie Dong, Xiang Chen, Jie Li, Peng Liu, Shuo Hu, Cong Peng

**Affiliations:** 1 The Department of Dermatology, Xiangya Hospital, Central South University, Changsha, 410000 Hunan China; 2 Department of Nuclear Medicine, Xiangya Hospital, Central South University, No. 87 Xiangya Road, Changsha, 410008 Hunan China; 3 Hunan Key Laboratory of Skin Cancer and Psoriasis, Hunan Engineering Research Center of Skin Health and Disease, Xiangya Hospital, Changsha, 410000 Hunan China; 4 Furong Laboratory, Changsha, 410000 Hunan China; 5 National Engineering Research Center of Personalized Diagnostic and Therapeutic Technology, Changsha, 410000 Hunan China; 6 National Clinical Research Center for Geriatric Diseases, Xiangya Hospital, Changsha, 410000 Hunan China; 7 Key Laboratory of Biological Nanotechnology, NHC., No. 87 Xiangya Road, Changsha Hunan, 410008 China; 8 Xiangya School of Pharmaceutical Sciences, Central South University, Changsha, 410013 Hunan China

**Keywords:** Atopic dermatitis, Calcium phosphate nanoparticles, siRNA, Transdermal delivery, Cuproptosis, Pyroptosis

## Abstract

**Background:**

Atopic dermatitis (AD) is a common chronic inflammatory skin disorder characterized by epidermal barrier dysfunction and immune dysregulation, yet effective long-term therapies are limited. Although regulated cell death has been linked to AD, the involvement of copper-dependent cell death (cuproptosis) and its therapeutic relevance in AD have not been explored.

**Results:**

Herein, we identify aberrant epidermal upregulation of the copper transporter SLC31A1 as a driver of copper overload and cuproptosis in keratinocytes, which in turn promotes GSDMA-dependent pyroptosis through an α-ketoglutarate (α-KG)/H3K9me3 epigenetic mechanism. To target this pathway, we developed a dual-functional microneedle system composed of calcium phosphate nanoparticles delivering Slc31a1 siRNA and embedded within *Bletilla striata* polysaccharide microneedles (CaP-siSlc31a1@BSP). This platform enables efficient transdermal gene silencing while BSP simultaneously suppresses STAT3/GSDMA signaling and inflammation. In MC903-induced AD-like mice, CaP-siSlc31a1@BSP markedly alleviated skin inflammation, epidermal hyperplasia and pruritus, accompanied by reduced Th2/Th17 responses.

**Conclusions:**

Our study reveals a previously unrecognized cuproptosis-pyroptosis axis in AD and establishes SLC31A1 as a promising therapeutic target. The CaP-siSlc31a1@BSP microneedle offers a synergistic drug-gene transdermal strategy with strong potential for AD treatment.

**Graphical Abstract:**

**Figure:**
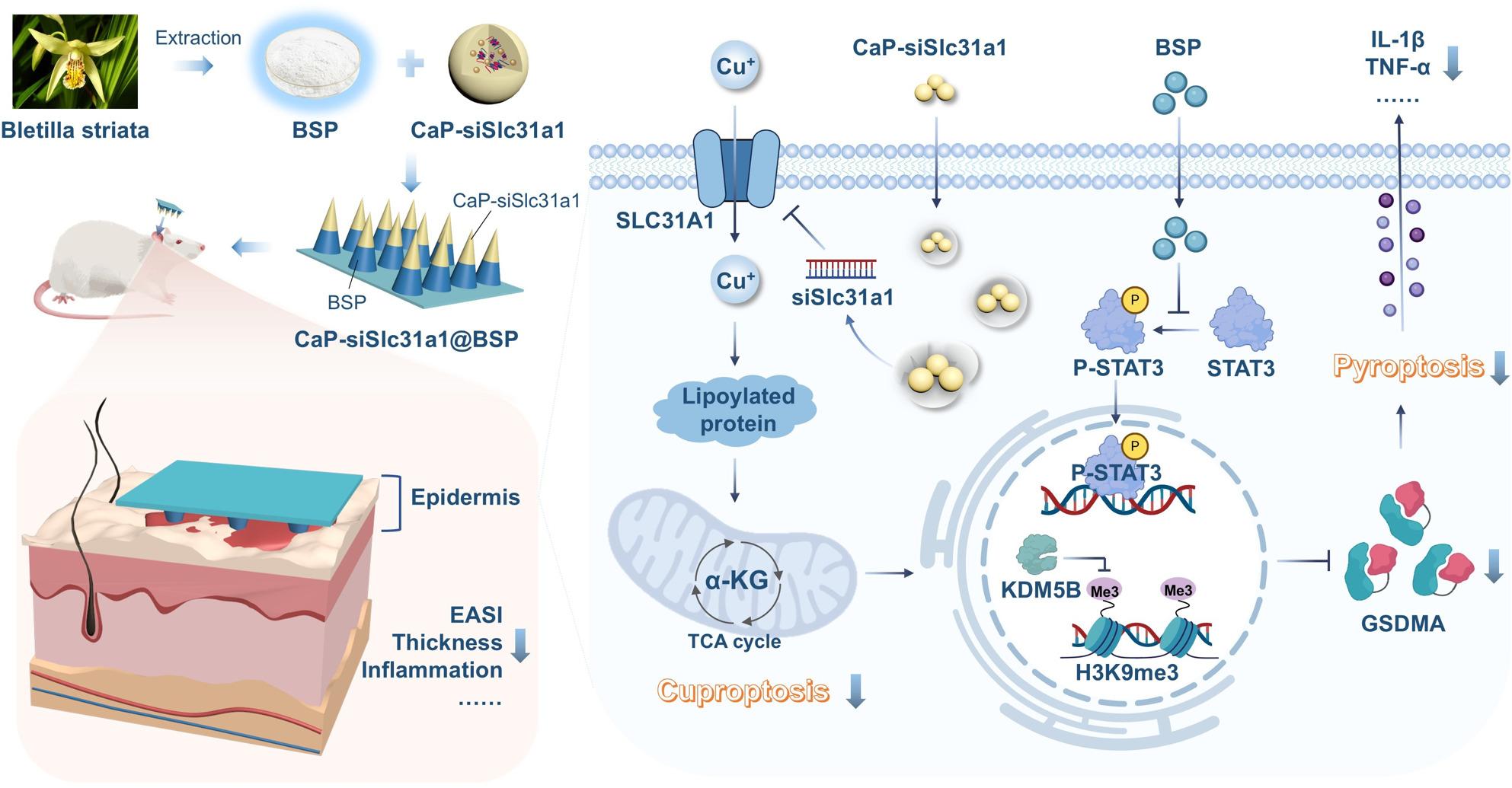
Upregulated SLC31A1 in lesional epidermis triggers cuproptosis-dependent α-KG accumulation, which epigenetically remodels chromatin (H3K9me3) to hyperactivate GSDMA transcription. The CaP-siSlc31a1@BSP microneedle system breaks this cycle via a dual-target strategy: (1) silencing SLC31A1 to block cuproptosis-induced metabolic stress, and (2) suppressing STAT3 phosphorylation to shut down transcriptional signaling. This combinatorial approach achieves superior therapeutic precision by simultaneously targeting metabolic-epigenetic crosstalk and cytokine signaling pathways.

**Supplementary Information:**

The online version contains supplementary material available at 10.1186/s12951-026-04533-9.

## Introduction

Atopic dermatitis (AD) is one of the most common chronic inflammatory skin disorders, affecting nearly all age groups, with a global prevalence of approximately 10% in adults and up to 20% in children [1]. Beyond the visible symptoms such as erythema, edema, desquamation, and lichenification, patients often suffer from intense pruritus, recurrent infections, and significant psychosocial distress, collectively leading to impaired quality of life [2]. The pathogenesis of AD is multifactorial, involving epidermal barrier dysfunction, genetic predisposition, environmental triggers, and immune dysregulation. A hallmark feature is the aberrant activation of type 2 immune responses, characterized by elevated Th2 cytokines, eosinophilia, and increased serum IgE [3]. Current therapies rely heavily on topical corticosteroids, calcineurin inhibitors, systemic immunosuppressants, and biologics targeting cytokines such as IL-4 and IL-13. Although effective in some cases, these treatments face limitations including glucocorticoid resistance, adverse effects associated with long-term use, high costs, and suboptimal patient compliance [4, 5]. These challenges underscore the urgent need to develop safe and effective therapeutic strategies that can precisely modulate disease-relevant pathways and provide long-term disease control.

Copper metabolism has recently emerged as an important regulator of cellular homeostasis and inflammatory processes. Excess copper induces mitochondrial protein aggregation and drives a distinct form of regulated cell death known as cuproptosis [6, 7]. Unlike apoptosis, necroptosis, or ferroptosis, cuproptosis is mediated by copper binding to lipoylated proteins in the tricarboxylic acid (TCA) cycle, leading to proteotoxic stress and cell death [8]. The solute carrier family 31 member 1 (SLC31A1), the principal transporter mediating copper influx, is a critical determinant of intracellular copper accumulation and thereby a central regulator of cuproptosis [9]. Recent studies have implicated dysregulated copper metabolism and cuproptosis in inflammatory and autoimmune disorders such as rheumatoid arthritis, Crohn’s disease, and osteoarthritis [10–12]. Nevertheless, their role in AD has not yet been elucidated. In parallel, increasing evidence suggests that pyroptosis, an inflammatory form of cell death executed by the gasdermin family, contributes to skin inflammation and barrier disruption in AD. Our preliminary studies indicate that excessive SLC31A1 expression may link copper-driven metabolic stress with pyroptotic pathways in keratinocytes (KCs), suggesting a potential mechanistic crosstalk between cuproptosis and pyroptosis in AD pathogenesis.

Gene silencing technologies provide powerful tools to dissect and therapeutically modulate such disease-relevant pathways [13]. Small interfering RNA (siRNA) has attracted particular attention due to its high specificity in degrading target mRNAs, thereby offering precise regulation of genes such as SLC31A1 [14]. However, clinical translation of siRNA remains hindered by several key barriers, including susceptibility to nuclease degradation, limited stability in biological fluids, poor bioavailability, and inefficient cellular uptake [15–18]. Although lipid nanoparticles and viral vectors have been explored as siRNA carriers, issues of toxicity, immunogenicity, and limited tissue specificity persist. In this context, calcium phosphate (CaP) nanoparticles have re-emerged as an attractive alternative. Based on biomineralization principles, CaP nanoparticles possess excellent biocompatibility, biodegradability, and pH-responsive release properties [19]. They have a decades-long history as safe transfection agents and have demonstrated superior performance in gene delivery [20]. These features highlight CaP nanoparticles as a promising platform for siRNA delivery to regulate SLC31A1 expression and, in turn, modulate copper-dependent cell death pathways in AD.

Nevertheless, the therapeutic application of CaP nanoparticles in dermatology remains limited by their poor penetration through the stratum corneum, the major barrier of the skin [21]. To overcome this limitation, microneedle (MN) technology offers a minimally invasive solution for transdermal drug delivery [22]. MNs create transient microchannels across the epidermal barrier, facilitating localized delivery of therapeutic cargos into the dermis with minimal discomfort [23, 24]. Among potential MN matrix materials, *Bletilla striata* polysaccharide (BSP), a natural polymer derived from a traditional medicinal herb, has attracted increasing interest. BSP exhibits anti-inflammatory, antioxidant, and immunomodulatory activities and is both biocompatible and biodegradable, making it an excellent candidate for biomedical applications [25]. Our earlier work and that of others demonstrated that BSP-based microneedles can reduce pro-inflammatory cytokine expression and inhibit STAT3-mediated signaling in hypertrophic scars, suggesting both intrinsic therapeutic potential and suitability as a microneedle matrix [26]. Importantly, BSP MNs not only provide structural support for effective CaP nanoparticle delivery but may also synergize with gene therapy by directly suppressing inflammation-related signaling pathways.

**Scheme 1.**
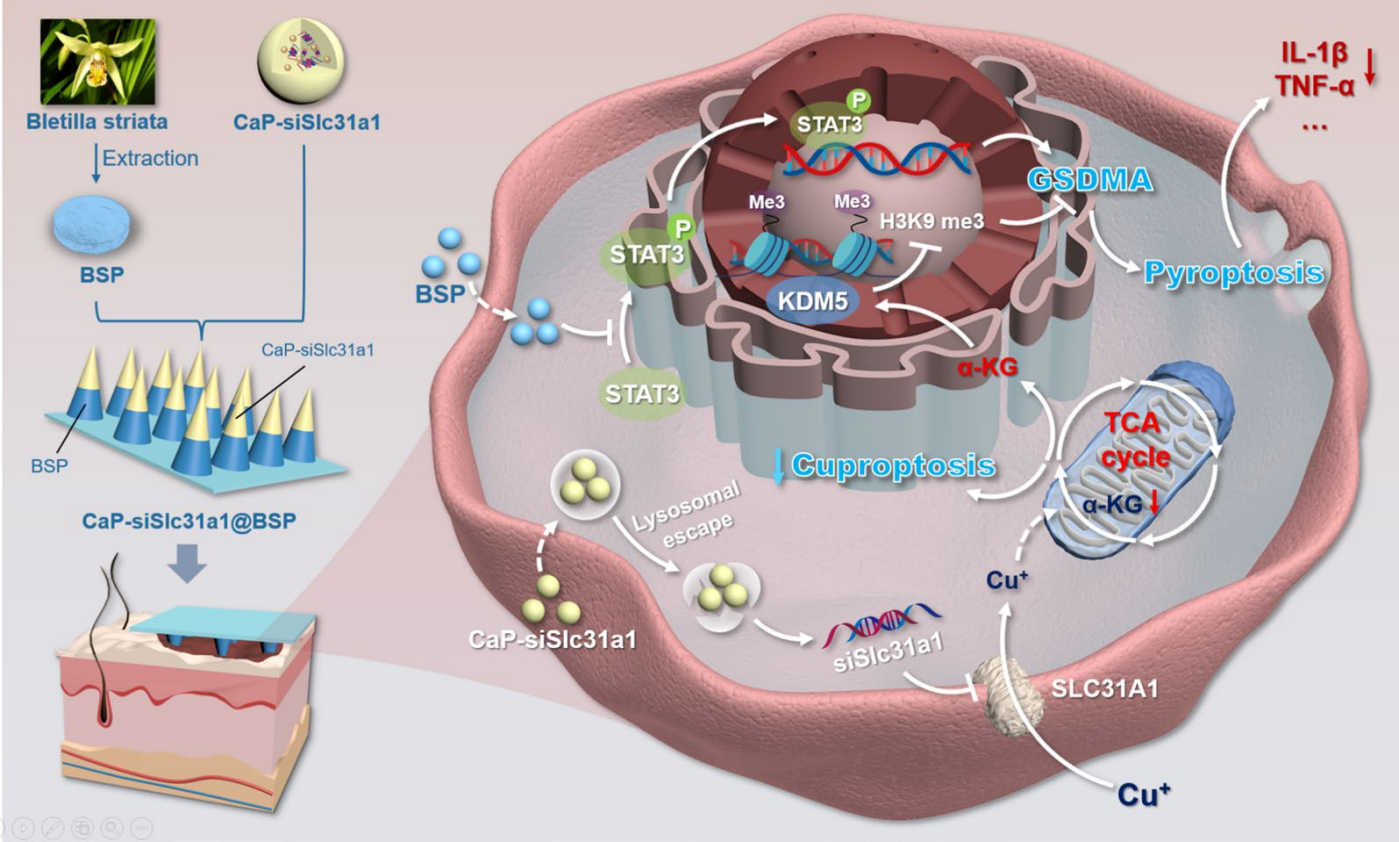
Schematic illustration of the synergistic therapeutic effect of CaP-siSlc31a1@BSP microneedles on AD. In the epidermis of MC903-induced AD-like mice, elevated SLC31A1 expression mediates cuproptosis, leading to α-KG accumulation. This accumulation enhances GSDMA transcription through H3K9me3, thereby exacerbating AD-like inflammation. CaP-siSlc31a1@BSP microneedles exert dual effects: CaP-siSlc31a1 suppresses epidermal SLC31A1 expression, while BSP inhibits STAT3 phosphorylation-mediated signaling to downregulate GSDMA transcription. Together, these actions synergistically enhance the precision and efficacy of AD therapy

Herein, we present the rational design of a dual-functional therapeutic platform, CaP-siSlc31a1@BSP microneedle, for the treatment of AD. This system integrates the gene-silencing capability of siSlc31a1-loaded CaP nanoparticles with the intrinsic anti-inflammatory properties of BSP-based MNs, enabling synergistic suppression of pathogenic pathways. Mechanistically, CaP-siSlc31a1 disrupts copper-driven cuproptosis and the subsequent transition to pyroptosis by silencing SLC31A1, while BSP concurrently inhibits STAT3/GSDMA-mediated pyroptosis. The MN platform ensures efficient, localized, and minimally invasive delivery into the skin, thereby enhancing therapeutic efficacy and minimizing systemic exposure. Collectively, this study not only identifies SLC31A1 as a previously unrecognized therapeutic target in AD but also introduces CaP-siSlc31a1@BSP as a novel synergistic drug-gene therapy strategy with strong potential for clinical translation (Scheme 1).

## Results and discussion

### SLC31A1-mediated cuproptosis is involved in the pathogenesis of AD

**Fig. 1:**
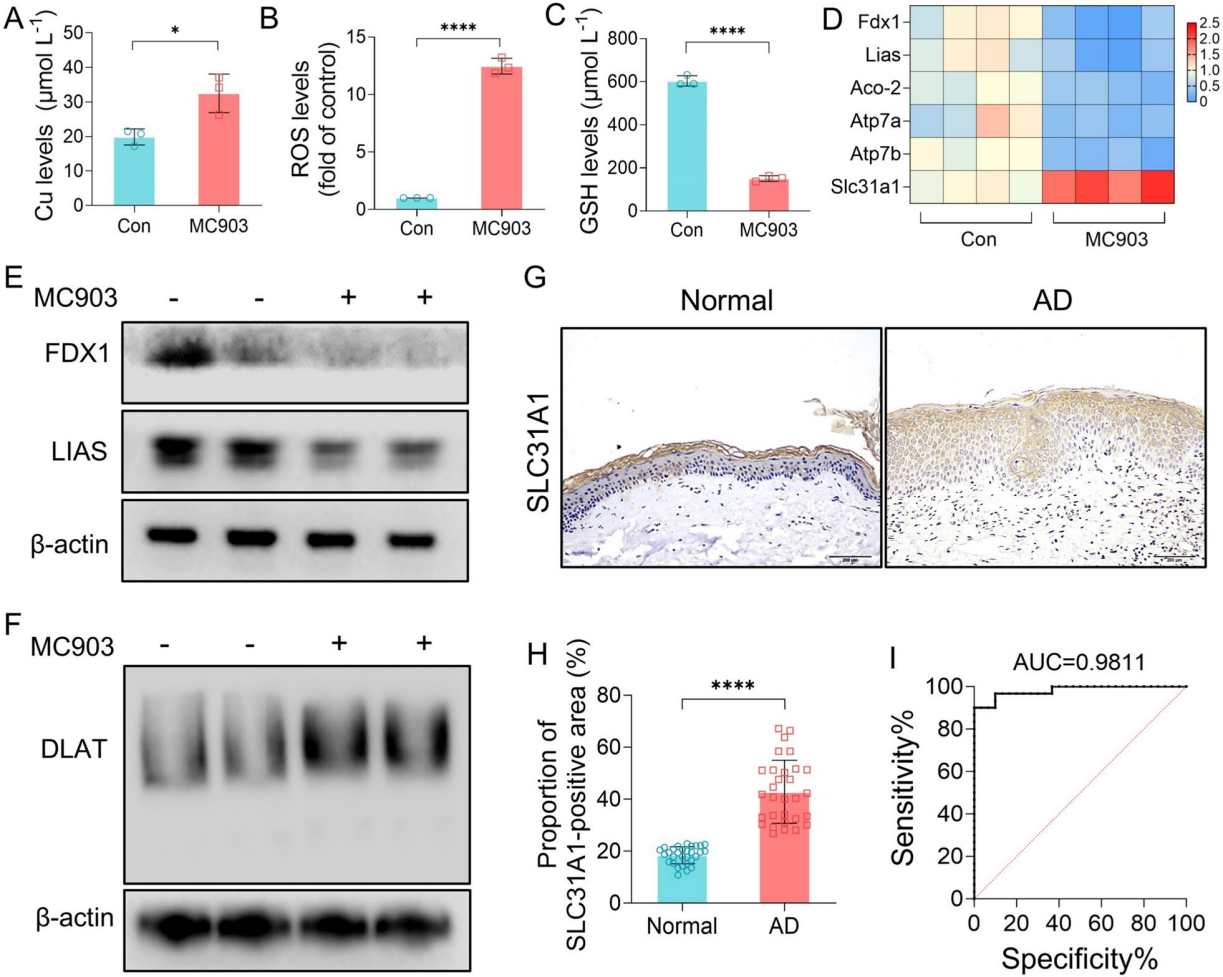
SLC31A1-mediated cuproptosis is involved in the pathogenesis of AD. Levels of (**A**) copper (Cu), (**B**) ROS, and (**C**) GSH in the epidermis of MC903-induced AD-like mice (n = 3). (**D**) Heatmap displaying the relative mRNA expression levels of *Fdx1*, *Lias*, *Aco-2*, *Atp7a/b*, and *Slc31a1* in the epidermis of MC903-induced AD-like mice (n = 4). Representative immunoblot bands for (**E**) FDX1 and LIAS and (**F**) DLAT in skin tissue from MC903-induced AD-like mice and control mice. (**G**) Immunohistochemical detection of SLC31A1 expression in skin tissues from healthy individuals and AD patients. One representative sample per group is shown. Scale bar: 200 μm. (**H**) Quantification of SLC31A1 expression levels in the data presented in (n = 30). (**I**) ROC curve analysis evaluating the correlation between epidermal SLC31A1 expression, as determined by immunohistochemistry, and AD in skin tissues from healthy individuals and AD patients (n = 30). Data represent SD ± mean. ns, not significant; *p < 0.05; **p < 0.01; ***p < 0.001; ****p < 0.0001

To investigate the potential involvement of cuproptosis in AD, we established a mouse model using MC903-induced chronic dermatitis. Repeated topical application to the ears over 12 days (with a 2-day rest on days 6-7) successfully induced hallmark AD-like features (Figure S1). Analysis of lesional skin revealed a significant increase in epidermal copper ion levels, accompanied by elevated reactive oxygen species (ROS) and a marked depletion of glutathione (GSH) (Fig. 1A-C), indicating an imbalance in redox homeostasis driven by copper overload. Further molecular analysis showed upregulation of the copper influx transporter SLC31A1, along with downregulation of copper efflux transporters (ATP7A, ATP7B) and iron-sulfur cluster-related proteins (FDX1, LIAS, ACO-2) (Fig. 1D, 1E). Notably, Dihydrolipoamide S-acetyltransferase (DLAT) exhibited oligomerization (Fig. 1F), a well-established pathological hallmark of copper-dependent metabolic dysregulation.

To validate these observations in humans, we performed immunohistochemistry on skin samples from AD patients and healthy controls. Epidermal expression of SLC31A1 was markedly higher in AD patients (Fig. 1G, 1H). Receiver operating characteristic (ROC) curve analysis further demonstrated a strong association between SLC31A1 expression and AD (AUC = 0.9811, Fig. 1I), supporting its potential as a diagnostic biomarker. Taken together, these results indicated that epidermal copper homeostasis is profoundly disrupted in AD. The upregulation of SLC31A1 and concomitant metabolic stress showed that cuproptosis may contribute to AD pathogenesis, both by amplifying oxidative stress and impairing mitochondrial integrity.

### Preparation and intracellular uptake of CaP-siSlc31a1

**Fig. 2:**
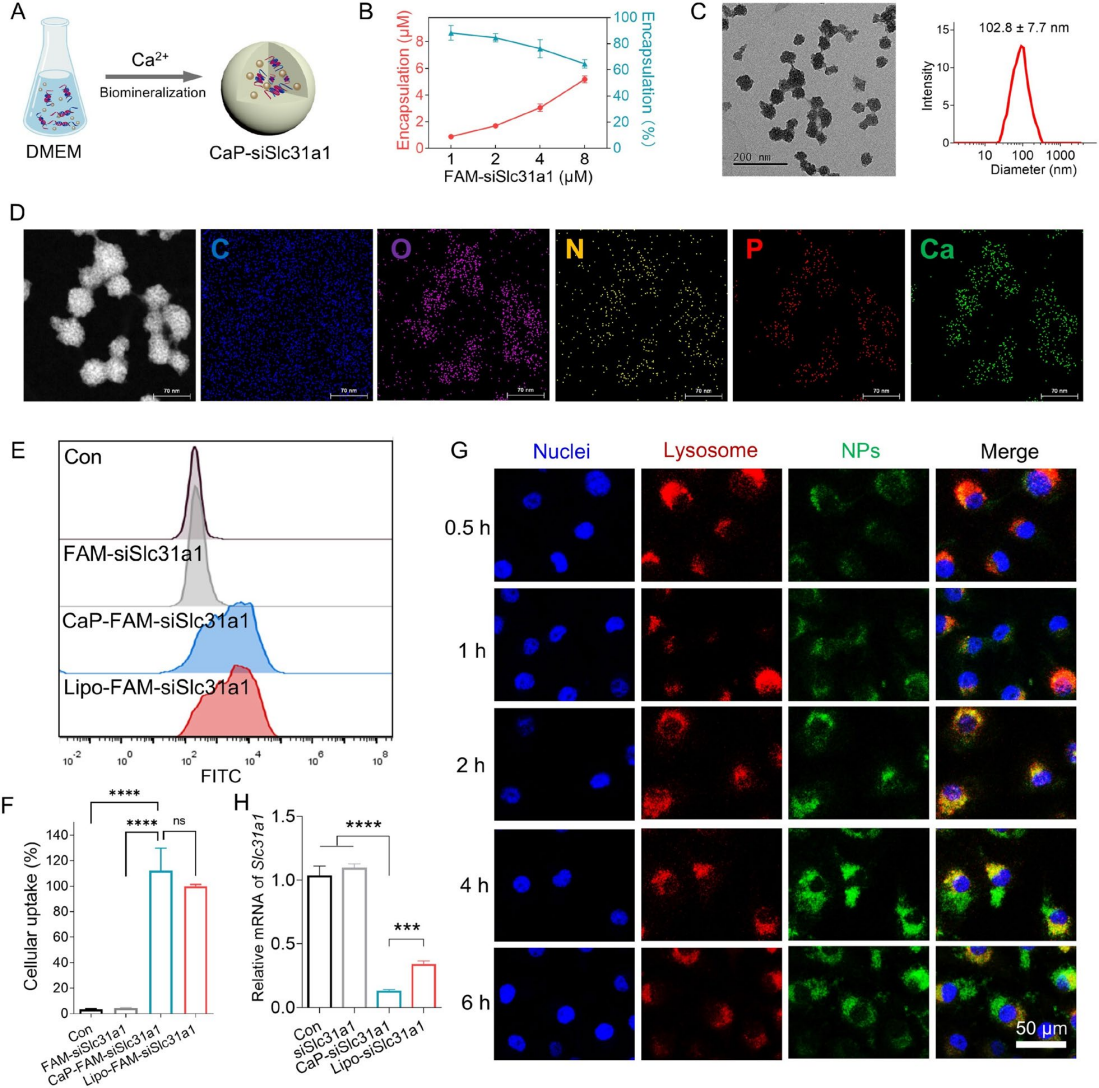
Preparation and intracellular uptake of CaP-siSlc31a1. (**A**) Scheme for the preparation of CaP-siSlc31a1. (**B**) The encapsulation capacity and encapsulation efficiency of CaP-siSlc31a1 at different siSlc31a1 feeding concentrations. (**C**) TEM image, dynamic light scattering analysis and (**D**) elemental mapping images of CaP-siSlc31a1. (**E**) Flow cytometric analysis and (**F**) quantification of nanoparticles uptake in KCs. (**G**) Colocalization observation by CLSM of CaP-FAM-siSlc31a1 (green) and Lysotracker (red). (**H**) The expressions of *Slc31a1* in different groups. Data represent SD ± mean. ns, not significant; *p < 0.05; **p < 0.01; ***p < 0.001; ****p < 0.0001

Based on the above findings that implicate SLC31A1 in AD pathogenesis, we designed and fabricated a CaP nanosystem capable of delivering Slc31a1 siRNA (siSlc31a1) to regulate SLC31A1 expression. The siSlc31a1-loaded CaP nanoparticles (CaP-siSlc31a1) were prepared via a biomineralization method (Fig. 2A). Briefly, Dulbecco’s modified Eagle’s medium (DMEM), serving as a biomimetic extracellular fluid, was mixed with bovine serum albumin (BSA) and siSlc31a1. The mixture was subsequently added to a CaCl_2_ solution to initiate in situ biomineralization. The Ca^2+^ reacted with anionic phosphate groups present in DMEM, resulting in the nucleation of calcium phosphate nanocrystals. Over time, these crystallites underwent oriented attachment and matured into dense, stable CaP-siSlc31a1 nanoparticles. To assess the drug loading capacity, CaP-FAM-siSlc31a1 nanoparticles were fabricated using FAM-labeled siSlc31a1 (FAM-siSlc31a1) at various feeding concentrations, and the amount of loaded siSlc31a1 was quantified using a fluorospectrophotometer. The encapsulation capacity of siSlc31a1 within the CaP increased with the initial feeding concentration of siSlc31a1 in the reaction mixture. In contrast, the encapsulation efficiency, defined as the percentage of siSlc31a1 incorporated into the nanoparticles relative to the total amount added, decreased at higher siSlc31a1 concentrations (Fig. 2B). Based on these measurements, an optimal siSlc31a1 concentration of 2 μM was identified. The obtained CaP-siSlc31a1 nanoparticles exhibited a uniform spherical morphology with an average diameter of approximately 102.8 nm, as determined by both transmission electron microscopy (TEM) and dynamic light scattering (DLS) (Fig. 2C). Elemental mapping confirmed the presence of C, O, N, P, and Ca within the nanostructure (Fig. 2D).

Then, the performances of CaP-siSlc31a1 inside mouse primary KCs to regulate siSlc31a1 expression were investigated. The uptake of CaP-siSlc31a1 nanoparticle by KCs was assessed by measuring fluorescence intensity via flow cytometry. KCs treated with CaP-FAM-siSlc31a1 demonstrated markedly enhanced fluorescence intensity compared to those treated with free FAM-siSlc31a1 (Fig. 2E, 2F). Notably, the internalization efficiency of CaP was comparable to that achieved with the commercial transfection reagent Lipofectamine 2000 (Lipo). Confocal laser scanning microscopy (CLSM) imaging further confirmed the efficient internalization of nanoparticles by KCs, as evidenced by a time-dependent increase in green fluorescence intensity within the cells (Fig. 2G). After successful internalization, the lysosomal escape associated with CaP-siSlc31a1 was further evaluated. Colocalization of green and red fluorescence signals indicated that the nanoparticles were primarily trapped within lysosomes. In contrast, after 4 h of incubation, a distinct separation between the nanoparticles and lysosomes was observed, accompanied by a significant decrease in Pearson’s correlation from 1 to 6 h (Figure S2), suggesting that FAM-siSlc31a1 had effectively escaped from the lysosomes. Owing to effective cellular uptake and timely lysosome escape, intracellular levels of *Slc31a1* were significantly downregulated in CaP-siSlc31a1 group (Fig. 2H). Notably, Slc31a1 expression was lower in the CaP-siSlc31a1 group compared to the Lipo-siSlc31a1 group, indicating that CaP-siSlc31a1 serves as a more efficient gene silencing platform for targeting Slc31a1.

### CaP-siSlc31a1 modulates epidermal cuproptosis-pyroptosis switch via regulation of GSDMA

**Fig. 3:**
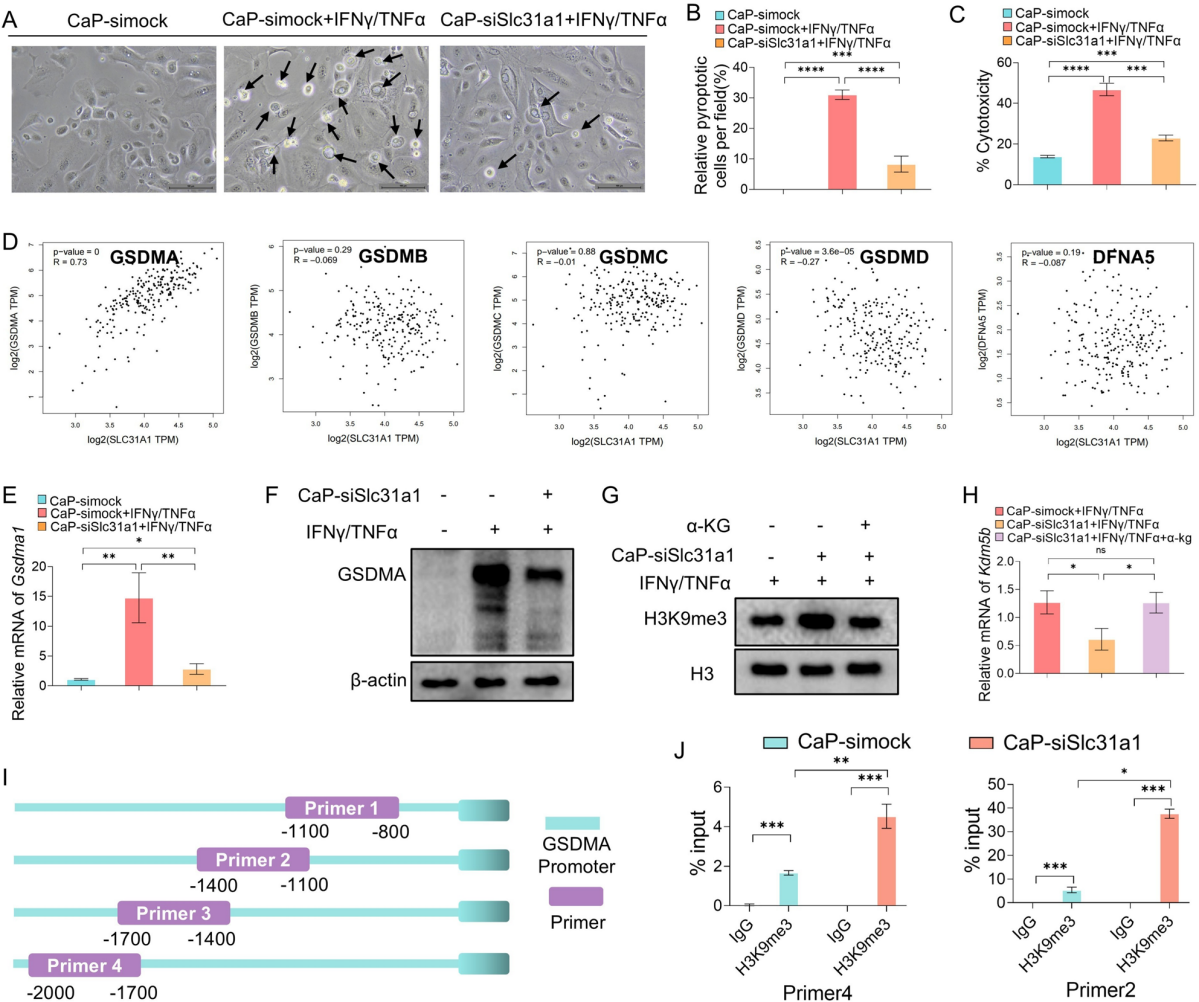
Epidermal SLC31A1 modulates the cuproptosis-pyroptosis switch via regulation of GSDMA. (**A**-**C**) Mouse primary KCs were treated with CaP-siSlc31a1 or CaP-simock for 48 h, followed by IFNγ/TNFα (10 ng mL^−1^) stimulation for 24 h. (A) Representative images showing dying cell morphology from three independent experiments; black arrows indicate swollen cells with large bubbles. Scale bar, 100 μm. (**B**) Quantification of pyroptotic cells per unit area (n = 3). (**C**) LDH release indicating cell death, shown as mean ± SD (n = 3). (**D**) GEPIA2 database analysis revealed a positive correlation between SLC31A1 and the pyroptosis-related gene GSDMA, but not with GSDMB, GSDMC, GSDMD, or GSDME. (**E**–**F**) Mouse primary KCs were treated with CaP-siSlc31a1 or CaP-simock for 48 h, followed by IFNγ/TNFα (10 ng mL^−1^) stimulation for 24 h. (**E**) Relative mRNA levels of *Gsdma1* (n = 3). (**F**) Representative immunoblot bands of GSDMA. (**G**-**H**) Mouse primary KCs were treated as indicated for 24 h. (**G**) Representative immunoblot bands of H3K9me3. (**H**) Relative mRNA levels of *Kdm5b* (n = 3). (**I**) Predicted H3K9me3 binding sites identified using the Cistrome Data Browser. (**J**) Mouse primary KCs were treated with CaP-siSlc31a1 or CaP-simock for 48 h. ChIP analysis of H3K9me3 occupancy at the GSDMA promoter; DNA was immunoprecipitated with an H3K9me3-specific antibody. Bars represent relative PCR product levels associated with H3K9me3 in the GSDMA promoter region. Data represent SD ± mean. ns, not significant; *p < 0.05; **p < 0.01; ***p < 0.001; ****p < 0.0001

To investigate the mechanism by which CaP-siSlc31a1 regulates AD pathogenesis in vitro, we established a cell model of the AD-related inflammatory microenvironment by exposing mouse primary KCs to interferon-γ (IFNγ) and tumor necrosis factor-α (TNFα) for 24 h, two cytokines known to contribute to AD development and severity. This stimulation significantly upregulated the mRNA expression of AD-associated mediators, including *Il1b, Il4, Il6, Il13, Il31, Cxcl3,* and *Ifng* (Figure S3), confirming the successful establishment of the model. Optical microscopy revealed that IFNγ/TNFα stimulation induced characteristic pyroptotic morphology, including balloon-like swelling, which was markedly attenuated by CaP-siSlc31a1 treatment compared with CaP-simock (Scrambled siRNA) (Fig. 3A, 3B). In line with this, lactate dehydrogenase (LDH), a well-established marker of cellular damage, inflammation, and pyroptosis [27], was significantly elevated in IFNγ/TNFα-stimulated KCs but substantially reduced following CaP-siSlc31a1 treatment (Fig. 3C). Gasdermins, pore-forming effector proteins that mediate membrane permeabilization and pyroptosis, were then investigated [28]. GEPIA2 database analysis revealed a positive correlation between SLC31A1 and GSDMA expression, but not with GSDMB, GSDMC, GSDMD, or GSDME (Fig. 3D). Notably, GSDMA is the predominant gasdermin in skin, and its activation through N-terminal domain release leads to pyroptosis [29, 30]. CaP-siSlc31a1 treatment significantly reduced both GSDMA mRNA and protein levels in IFNγ/TNFα-stimulated mouse primary KCs (Fig. 3E, 3F), implicating SLC31A1 as a regulator of GSDMA-mediated pyroptosis.

We then performed targeted metabolomics on skin lesions from MC903-induced mice models to explore CaP-siSlc31a1-mediated metabolic alterations in AD. Differential metabolite analysis revealed pronounced fluctuations in α-ketoglutarate (α-KG) (Figure S4), with KEGG pathway enrichment highlighting the TCA cycle as a central pathway (Figure S5). As a key metabolite in the mitochondrial TCA cycle, α-KG has been shown in previous studies to accumulate during cuproptosis [9, 31]. Oral administration of α-KG in MC903-induced mice markedly exacerbated erythema, scaling, and epidermal thickening (Figure S6), accompanied by elevated eczema area and severity index (EASI) scores (Figure S7). Furthermore, α-KG enhanced *Gsdma1* transcription and increased the mRNA levels of multiple pro-inflammatory cytokines and chemokines, including *Il1b, Il4, Il13, Il15, Cxcl1, Cxcl3, Tnf*, and *Tslp* (Figure S8). Mechanistically, elevated intracellular α-KG levels are known to activate histone lysine demethylase 5B (KDM5B), which reduces H3K9me3 levels through epigenetic modification [32]. Given that SLC31A1-mediated cuproptosis promotes α-KG accumulation, and that α-KG is abundant in AD, we hypothesize that SLC31A1 regulates GSDMA via α-KG-induced epigenetic remodeling. Supporting this, SLC31A1 downregulation using CaP-siSlc31a1 increased H3K9me3 levels in IFNγ/TNFα-stimulated mouse primary KCs, whereas α-KG supplementation reversed this effect (Fig. 3G). Consistently, CaP-siSlc31a1 reduced *Kdm5b* mRNA levels, which were restored by α-KG treatment (Fig. 3H). In addition, chromatin immunoprecipitation (ChIP) analysis using primers designed from the Cistrome Data Browser (http://cistrome.org/db/) revealed that H3K9me3 directly binds the GSDMA promoter (Fig. 3I, Table S2). CaP-siSlc31a1 treatment significantly increased this binding in mouse primary KCs (Fig. 3J). Together, these results indicated that CaP-siSlc31a1 inhibited KDM5B via α-KG, thereby increasing H3K9me3 and reducing GSDMA expression, ultimately preventing AD progression.

### Fabrication and characterization of CaP-siSlc31a1@BSP microneedle for aural application

**Fig. 4:**
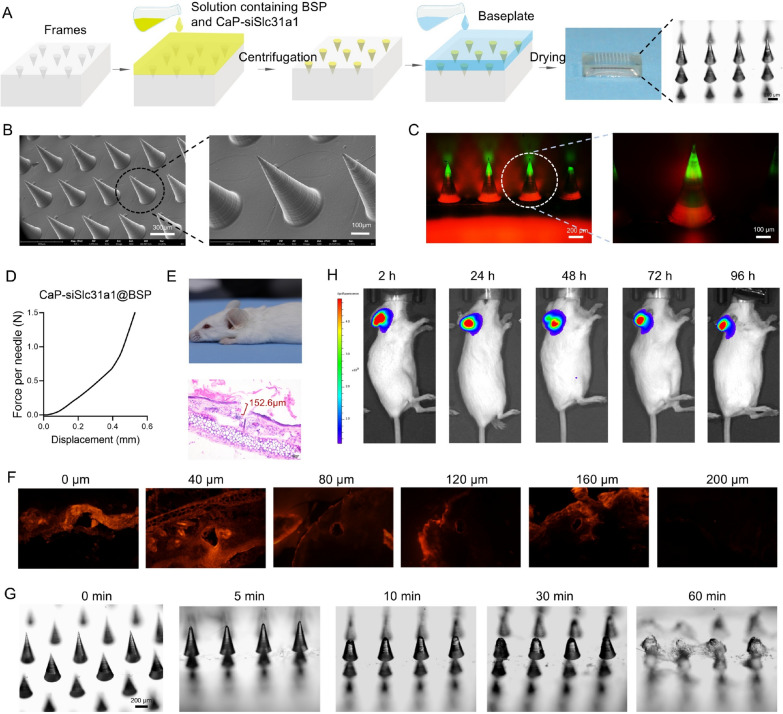
Fabrication and characterization of CaP-siSlc31a1@BSP microneedle for aural application. (**A**) Scheme for the fabrication of CaP-siSlc31a1@BSP. (**B**) SEM and (**C**) fluorescence microscopy images of CaP-siSlc31a1@BSP. (**D**) Force–displacement profiles of CaP-siSlc31a1@BSP. (**E**) Representative photograph of a mouse and H&E-stained ear tissue after CaP-siSlc31a1@BSP insertion. (**F**) Cross-sectional images showing puncture holes at various depths in mouse skin produced by CaP-siSlc31a1@BSP. (**G**) Dissolution process of CaP-siSlc31a1@BSP in mouse ear skin. (**H**) Biodistribution of nanoparticles following topical application of CaP-siSlc31a1@BSP

Having confirmed the potential of CaP-siSlc31a1 for AD management, we next explored an appropriate administration strategy to deliver it to local lesions. Topical administration enables targeted delivery by directly accumulating therapeutic agents at the lesion site while minimizing systemic exposure, thereby potentially enhancing therapeutic efficacy for AD [33]. Recently, microneedles have emerged as an ideal transdermal delivery strategy, mechanically puncturing the stratum corneum to form microchannels that enable efficient therapeutic delivery. Encouraged by our previous studies [34], we designed dissolving BSP microneedles for the delivery of CaP-siSlc31a1. The CaP-siSlc31a1@BSP was fabricated using a casting method. Briefly, the CaP-siSlc31a1 nanoparticles containing 2 nmol of siSlc31a1 were dispersed in the BSP matrix to form a homogeneous pre-gel solution. This mixture was introduced into microneedle molds and centrifuged to shape the needle tips. Subsequently, a hyaluronic acid (HA) solution was added to form the backing layer. After drying and demolding, a 10 × 10 microneedle array with BSP-based tips containing CaP-siSlc31a1 and a HA backing layer was obtained (Fig. 4A). Scanning electron microscopy (SEM) revealed that the CaP-siSlc31a1@BSP microneedle exhibited a smooth surface and well-preserved cone-shaped morphology, with needle tips uniformly integrated into the base substrate (Fig. 4B). The presence of C, O, N, Ca and P elements within the CaP-siSlc31a1@BSP was confirmed by element mapping analysis (Figure S9). To further visualization, the FAM-labeled nanoparticles and Rhodamine B-marked needles were incorporated into the microneedle structure and observed using fluorescence microscopy. The results showed that that CaP-siSlc31a1@BSP was effectively concentrated within the microneedle tips, which is crucial to allow for superior transdermal drug delivery (Fig. 4C).

Previous studies have indicated that a microneedle hardness in the range of 0.1-0.2 N/needle is sufficient to facilitate penetration through the stratum corneum [35, 36]. Both CaP-siSlc31a1@BSP (Fig. 4D) and BSP microneedles (Figure S10) exhibited mechanical strengths that significantly exceeded the minimum force required for dermal penetration. This was further evidenced by hematoxylin–eosin (H&E) staining of skin sections, which revealed distinct puncture holes indicative of the high mechanical integrity of CaP-siSlc31a1@BSP (Fig. 4E). Additionally, the puncture depth, targeted at 200 μm, was determined by analyzing the fluorescence distribution within the skin tissue (Fig. 4F). The dissolution rate following dermal penetration, a critical factor influencing nanoparticle release and diffusion, was also assessed. The results revealed that the tips of the CaP-siSlc31a1@BSP microneedle began dissolving within 10 min post-insertion and were completely dissolved within 60 min (Fig. 4G). Prior to applying the microneedles for AD, we conducted a preliminary investigation of the biodistribution of CaP-siSlc31a1@BSP in an AD-like mouse model. For this purpose, CaP-siSlc31a1@BSP was topically applied to the ear skin of mice, and subsequent in vitro analyses showed that the fluorescence intensity remained robust up to 96 h post-application, suggesting effective accumulation of CaP-siSlc31a1 within the skin (Fig. 4H). These results underscore the potential of CaP-siSlc31a1@BSP as a therapeutic agent for dermatological conditions such as AD.

### BSP inhibit the progression of AD by modulating the STAT3/GSDMA axis

**Fig. 5:**
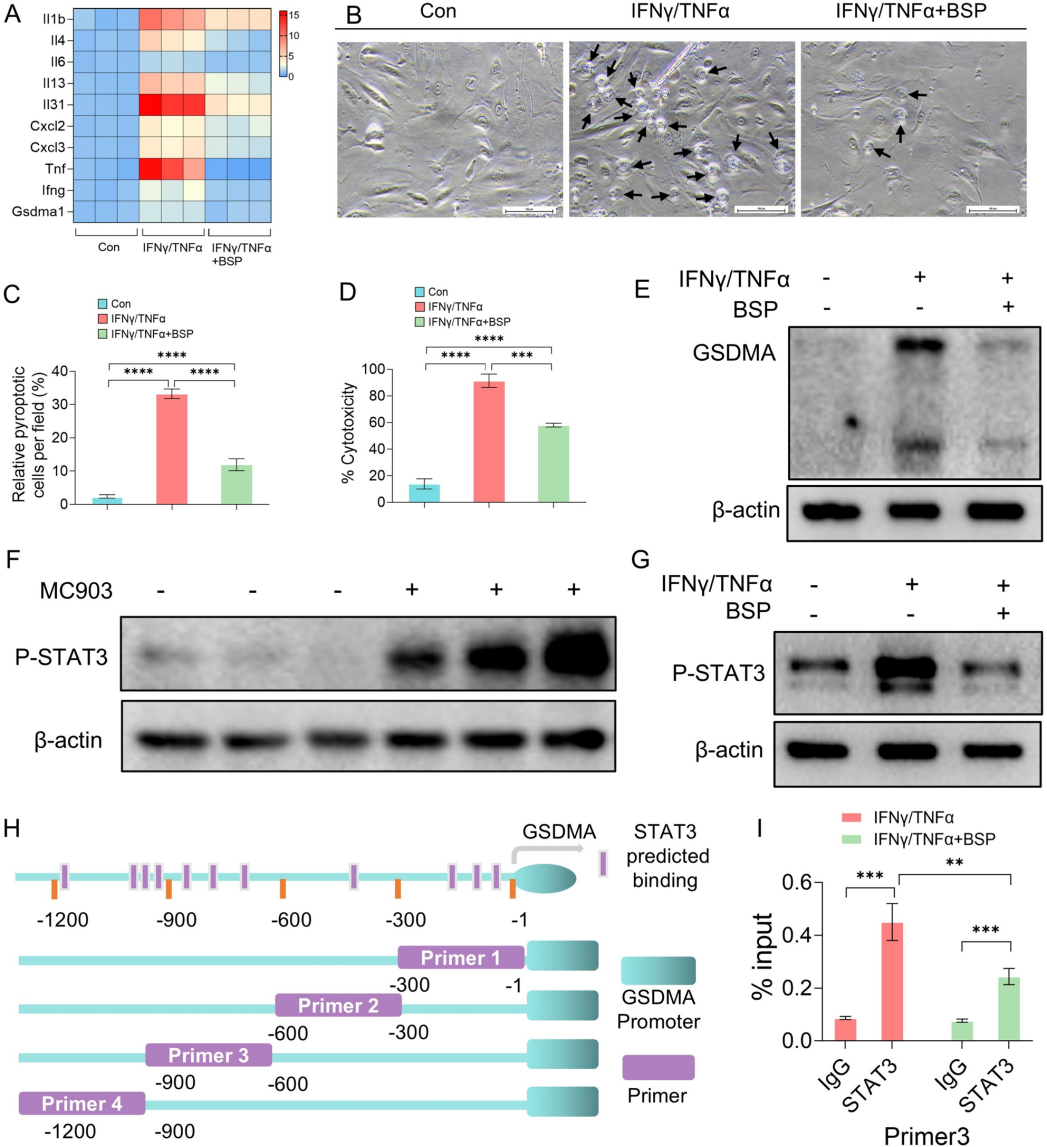
BSP inhibit the progression of AD by modulating the STAT3/GSDMA axis. (**A**) Relative mRNA levels of cytokines and chemokines in mouse primary KCs stimulated with IFNγ/TNFα (10 ng mL^−1^) or IFNγ/TNFα + BSP (10 mg mL^−1^) for 24 h (n = 3). (**B**-**D**) Assessment of pyroptotic cell morphology and quantification in KCs subjected to the same stimulation. (**B**) Representative images of dying cell morphology of three independent experiments; black arrows indicate cell swelling with large bubbles. Scale bar, 100 μm. (**C**) Quantification of pyroptotic cells per unit area (n = 3). (**D**) LDH-released cell death is shown as mean ± SD of n = 3 independent experiments. (**E**) Representative Western blot showing GSDMA protein levels in KCs treated as above for 24 h. (**F**) Representative Western blot showing P-STAT3 levels in skin lesions from MC903-induced AD-like mice models (n = 3). (**G**) Representative Western blot showing P-STAT3 levels in KCs treated as above for 24 h. (**H**) Predicted STAT3 binding sites identified using the JASPAR database. (**I**) ChIP analysis of STAT3 binding to the GSDMA promoter in mouse primary KCs stimulated with IFNγ/TNFα (10 ng mL^−1^) or IFNγ/TNFα + BSP (10 mg mL^−1^) for 24 h. DNA was immunoprecipitated using a specific STAT3 antibody. Bars represent relative levels of PCR products associated with STAT3 in the GSDMA promoter region. Data represent SD ± mean. ns, not significant; *p < 0.05; **p < 0.01; ***p < 0.001; ****p < 0.0001

Before the in vivo application of CaP-siSlc31a1@BSP, we investigated the therapeutic mechanisms of BSP in AD. BSP is recognized for its anti-inflammatory, antioxidant, and antibacterial properties [25]. To directly assess whether BSP inhibits AD-related inflammatory responses, we treated IFNγ/TNFα-stimulated mouse primary KCs with BSP, which significantly reduced the expression of the pyroptosis-related gene *Gsdma1* and Th2/Th17 cytokines (Fig. 5A). Morphological analysis of KCs pyroptosis by optical microscope, along with LDH release assays, further confirmed that BSP markedly suppressed pyroptosis under AD-like conditions (Fig. 5B-D). Western blot analysis revealed that BSP downregulated GSDMA and N-GSDMA protein expression (Fig. 5E), suggesting that its inhibitory effect on pyroptosis-related inflammation is mediated by suppression of GSDMA in KCs.

In addition, phosphorylated STAT3 (P-STAT3) plays a central role in immune dysregulation and skin barrier dysfunction in AD, and BSP targeting this pathway, such as JAK inhibitors [37, 38], have been intensively investigated. In line with this, our MC903-induced AD-like mice model showed significant P-STAT3 upregulation (Fig. 5F). Notably, BSP treatment markedly suppressed IFNγ/TNFα-induced P-STAT3 expression in KCs (Fig. 5G). Since STAT3 is a transcription factor that regulates pyroptosis-related genes, including *GSDMC* and *GSDME* [39], we hypothesized that BSP may inhibit *Gsdma* transcription by modulating STAT3 phosphorylation. To test this, we conducted ChIP assays to evaluate STAT3 binding at the GSDMA promoter region in KCs. Binding site predictions using the JASPAR database (https://jaspar.elixir.no/) guided the design of four primer pairs spanning the 1200 bp to 1 bp upstream region of the GSDMA promoter (Fig. 5H). Indeed, STAT3 was enriched at the GSDMA promoter in IFNγ/TNFα-stimulated mouse primary KCs, whereas BSP treatment abolished this interaction (Fig. 5I). In summary, BSP mitigates pyroptosis-associated inflammation and attenuates AD progression by suppressing STAT3 phosphorylation, thereby reducing the transcriptional activation of GSDMA.

### CaP-siSlc31a1@BSP significantly ameliorated MC903-induced AD-like skin inflammation.

**Fig. 6:**
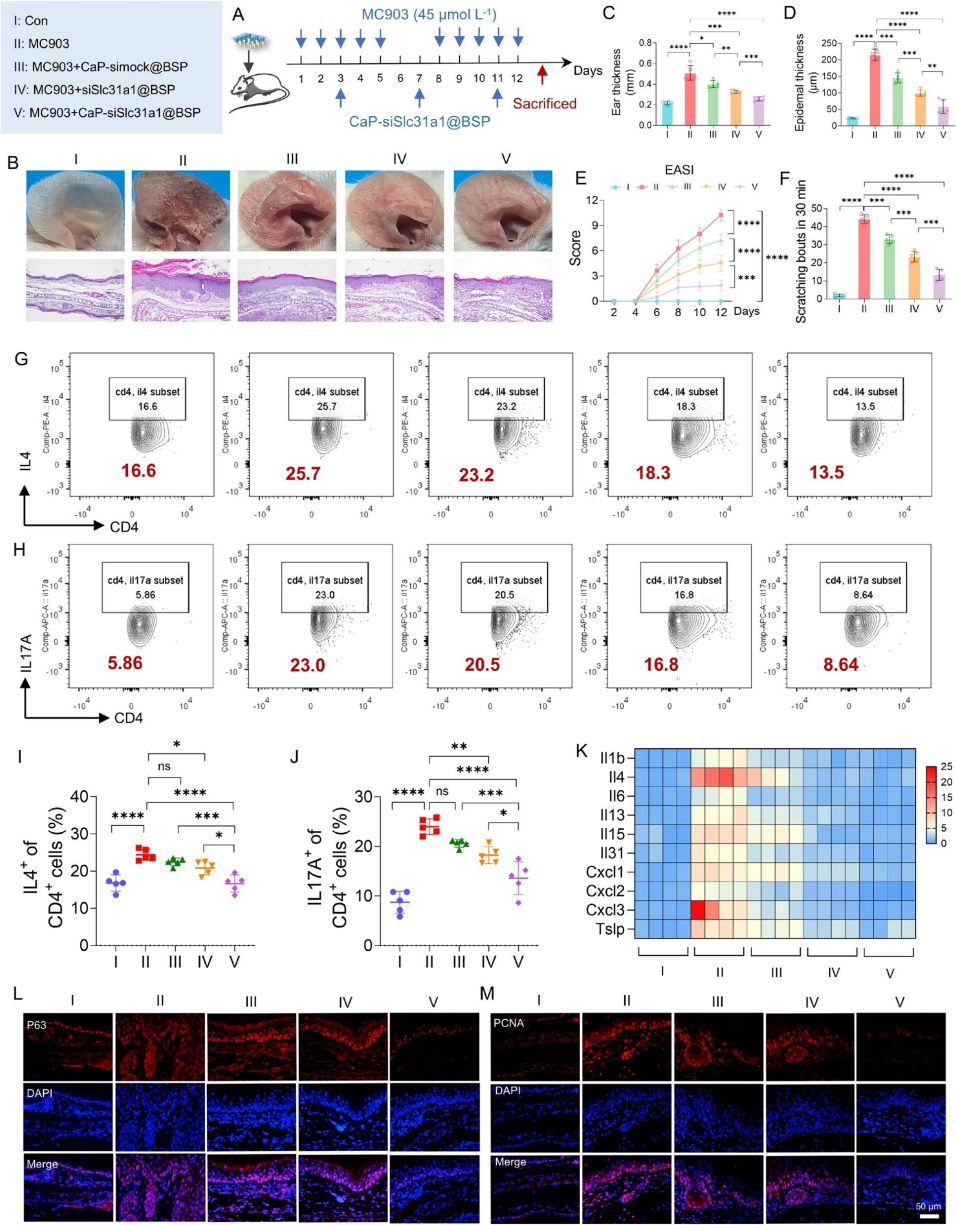
CaP-siSlc31a1@BSP significantly ameliorated MC903-induced AD-like skin inflammation. (**A**) Schematic illustration of AD induction in mice using MC903 and subsequent treatment with microneedles. (**B**) Representative images of mice and macroscopic views of ear skin stained with H&E. One representative mouse per group is shown (n = 5). Scale bar: 100 μm. (**C**) Statistical analysis of ear thickness and (**D**) epidermal thickness in mice following designated treatments. (**E**) Severity scores of mice, measured biweekly during the 12-day treatment period following designated interventions. (**F**) Scratching bouts within a 30-min period on day 12 following designated treatments. (**G**-**H**) Representative flow cytometry panels for quantifying IL4^+^ CD4^+^ and IL17A^+^ CD4^+^ cells after MC903 induction (n = 5). (**I**-**J**) Statistical analysis of flow cytometry data. (**K**) Relative mRNA levels of cytokines and chemokines in lesional skin tissue following designated treatments (n = 4). (**L**-**M**) Immunofluorescence detection of P63 and PCNA expression in lesional skin tissue following designated treatments. One representative image is shown per group (n = 3). Scale bar: 50 μm. The codes denote the following: Ⅰ, normal mice; Ⅱ, MC903-induced mice; Ⅲ, MC903-induced mice treated with CaP-simock@BSP; Ⅳ, MC903-induced mice treated with siSlc31a1@BSP; Ⅴ, MC903-induced mice treated with CaP-siSlc31a1@BSP. Data represent SD ± mean. ns, not significant; *p < 0.05; **p < 0.01; ***p < 0.001; ****p < 0.0001

We next evaluated the in vivo therapeutic potential of CaP-siSlc31a1@BSP in an MC903-induced AD-like mouse model. On days 3, 7, and 11 post-induction, microneedles were applied to the ears of mice (Fig. 6A). The model group developed severe cutaneous inflammation, including erythema, edema, and desquamation. In contrast, all microneedle groups exhibited varying degrees of therapeutic efficacy, as reflected by phenotype and H&E staining (Fig. 6B). Among them, the CaP-siSlc31a1@BSP demonstrated the most robust anti-AD activity, with the greatest reductions in ear and epidermal thickness (Fig. 6C, 6D). The CaP-simock@BSP group also showed moderate improvement compared with the untreated model group, confirming the intrinsic anti-AD activity of BSP. Moreover, siSlc31a1-loaded microneedle without CaP (siSlc31a1@BSP) displayed limited therapeutic efficacy, likely due to the poor cellular uptake of free siRNA. To quantify therapeutic efficacy, we applied the EASI scoring system every other day, assessing erythema/hemorrhage, thickening/dryness, edema, and excoriation/erosion of mice ears. Scores were significantly reduced in the CaP-siSlc31a1@BSP microneedle group, indicating a superior ability to alleviate AD (Fig. 6E). Pruritus, a hallmark of AD, was also evaluated. CaP-simock@BSP and siSlc31a1@BSP provided moderate relief of scratching behavior, whereas CaP-siSlc31a1@BSP significantly attenuated scratching (Fig. 6F), demonstrating the synergistic anti-pruritic effect of siSlc31a1 and BSP in AD management. In addition, mice were assessed for body weight every day and no significant differences were observed between treatment groups and controls (Figure S11).

To further verify the therapeutic effect of CaP-siSlc31a1@BSP microneedles, mouse models with MC903-induced AD-like dermatitis were administered with CaP-siSlc31a1@BSP microneedles and the positive control drug crisaborole (CB), respectively. As a first-line topical medication for AD, CB possesses high safety and potent anti-inflammatory and antipruritic efficacy, but is limited by insufficient transdermal delivery efficiency and local adverse reactions including burning sensation upon topical administration [40, 41]. The results showed that the therapeutic effect and body weight maintenance in the CaP-siSlc31a1@BSP microneedle group were comparable to those in the CB-treated positive control group (Figure S12). These findings suggest that the microneedle formulation exhibits therapeutic potency equivalent to the first-line clinical drug, and its excellent transdermal delivery property circumvents the penetration defect of CB, providing a more favorable topical intervention strategy for the treatment of AD.

To elucidate the underlying mechanisms, we examined immune cell infiltration in lesional skin by flow cytometry. CaP-simock@BSP and siSlc31a1@BSP induced only a mild reduction in Th2 and Th17 cell populations, while CaP-siSlc31a1@BSP microneedle caused a marked decrease, confirming potent inhibition of type 2 immune responses characteristic of AD (Fig. 6G-J). In addition, qRT-PCR analysis showed that MC903 treatment significantly increased mRNA expression of inflammatory cytokines (*Il1b, Il4, Il6, Il13, Il15, Il31, Cxcl1, Cxcl2, Cxcl3,* and *Tslp*) in lesional skin, whereas CaP-siSlc31a1@BSP substantially reduced their expression (Fig. 6K). We further assessed pathological changes by immunofluorescence. P63, particularly the ΔNp63α subtype, is associated with hyperproliferation and inflammation, while PCNA serves as a marker of DNA synthesis and proliferation [42, 43]. Both P63 and PCNA were strongly upregulated in MC903-treated skin. CaP-simock@BSP modestly reduced their expression, whereas CaP-siSlc31a1@BSP induced pronounced inhibition (Fig. 6L, 6M, Figure S13). Collectively, these results indicated that BSP microneedle-mediated delivery of siSlc31a1 effectively silences SLC31A1, acts synergistically with the therapeutic activity of BSP, suppresses downstream inflammatory pathways, inhibits epidermal proliferation and alleviates AD-like skin inflammation, underscoring the potential of CaP-siSlc31a1@BSP microneedle as a promising percutaneous therapeutic platform for AD.

### Ameliorating AD-like dermatitis via the SLC31A1/α-KG/H3K9me3/GSDMA and STAT3/GSDMA signaling axes.

**Fig. 7:**
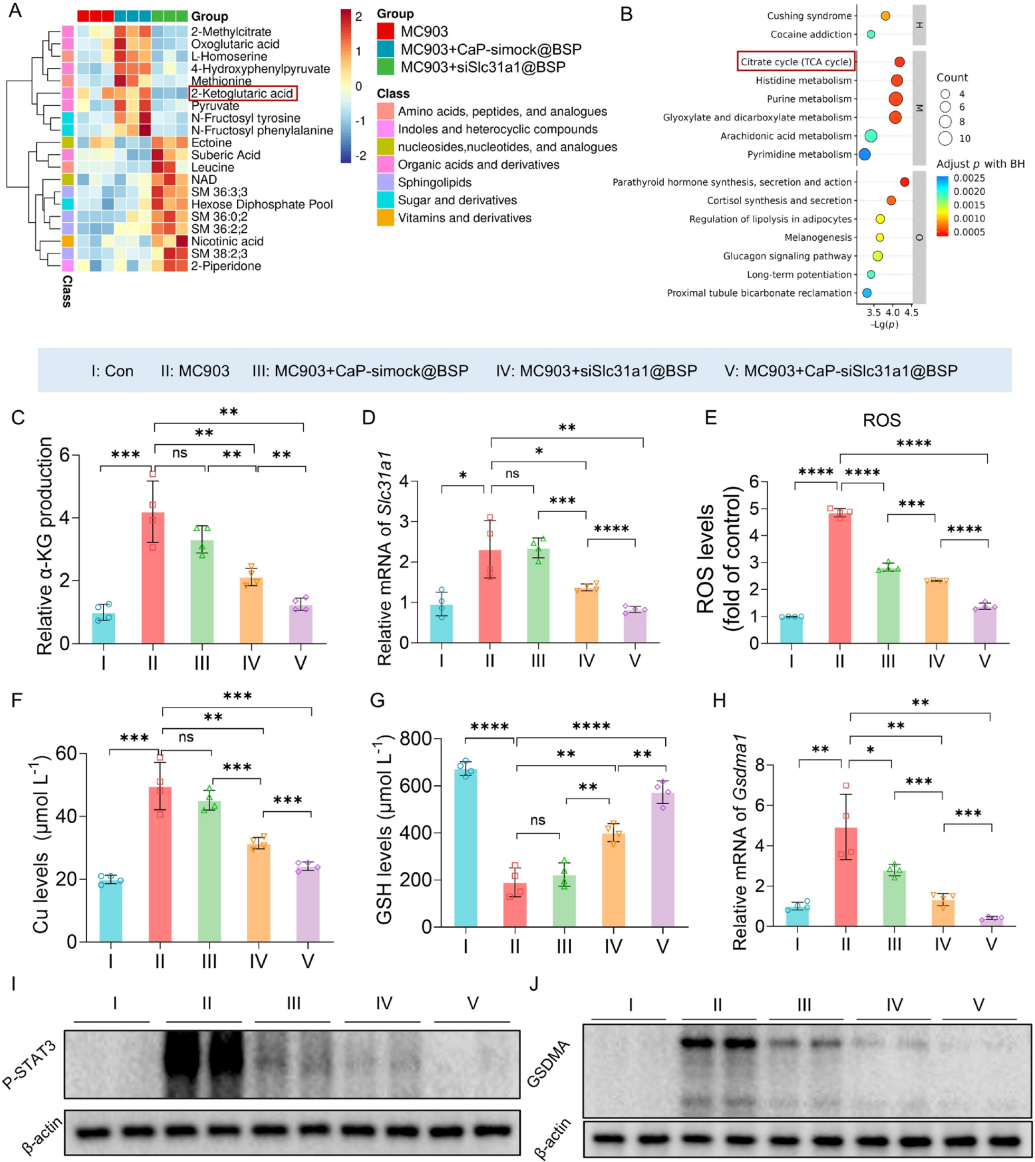
The CaP-siSlc31a1@BSP microneedle synergistically ameliorates AD-like dermatitis via the SLC31A1/α-KG/H3K9me3/GSDMA and STAT3/GSDMA signaling axes. (**A**) Heatmap displaying differential abundances of α-KG and other metabolites in mice skin tissues following designated treatments (n = 3). (**B**) Correlation analysis from targeted metabolomics showing the relationship between metabolites and pathways such as the TCA cycle in mice skin tissues after designated treatments (n = 3). (**C**) Relative concentration of α-KG in mice following designated treatments, as determined by an α-KG Assay kit (n = 4). (**D**) Relative mRNA levels of *Slc31a1* in skin lesions of mice after designated treatments (n = 4). (**E**–**G**) Levels of (**E**) ROS, (**F**) Cu, and (**G**) GSH in the epidermis of mice after different treatments (n = 4). (**H**) Relative mRNA levels of *Gsdma1* in skin lesions of mice after designated treatments (n = 4). (**I**) Representative immunoblot bands of P-STAT3 in skin lesions of mice after various treatments. (**J**) Representative immunoblot bands of GSDMA in skin lesions of mice after different treatments. The codes denote the following: Ⅰ, normal mice; Ⅱ, MC903-induced mice; Ⅲ, MC903-induced mice treated with CaP-simock@BSP; Ⅳ, MC903-induced mice treated with siSlc31a1@BSP; Ⅴ, MC903-induced mice treated with CaP-siSlc31a1@BSP. Data represent SD ± mean. ns, not significant; *p < 0.05; **p < 0.01; ***p < 0.001; ****p < 0.0001

Building on the significant therapeutic efficacy of the microneedle drug delivery system in the AD-like mouse model, we further explored its potential metabolic regulatory mechanisms using targeted metabolomics analysis. CaP-siSlc31a1@BSP treatment significantly reduced α-KG levels, which are tightly linked to the TCA cycle, whereas CaP-simock@BSP showed no such effect (Fig. 7A, 7B). This result was further confirmed using an α-KG Assay Kit (Fig. 7C). To clarify the molecular function of SLC31A1, we assessed the expression of related genes in the epidermis of treated AD-like mice. The qRT-PCR analysis revealed that CaP-siSlc31a1@BSP markedly decreased *Slc31a1* mRNA levels (Fig. 7D), consistent with the cell-based experiments, thereby confirming efficient target silencing. Analysis of lesional skin revealed that CaP-siSlc31a1@BSP significantly reduced ROS and copper ion levels, while notably increasing GSH content in the epidermis (Fig. 7E-G). These findings indicate that CaP-siSlc31a1@BSP ameliorates redox imbalance induced by copper overload. In addition, *Gsdma1* mRNA levels were significantly elevated in the epidermis of AD-like mice (Fig. 7H), whereas *Gsdma2*, *Gsdma3*, and other pyroptosis-related genes (*Gsdmc*, *Gsdmd*, *Gsdme*) remained unchanged (Figure S14). CaP-siSlc31a1@BSP strongly downregulated *Gsdma1* expression, while CaP-simock@BSP only partially alleviated this increase (Fig. 7H), highlighting the specific involvement of the SLC31A1/GSDMA axis. Furthermore, CaP-siSlc31a1@BSP significantly reduced P-STAT3 protein levels (Fig. 7I), leading to dual suppression of GSDMA expression (Fig. 7H, 7J). These findings suggest that upregulated SLC31A1 in AD drives α-KG accumulation, which subsequently activates KCs pyroptosis via GSDMA, thereby aggravating skin barrier disruption and inflammation. CaP-siSlc31a1@BSP microneedle mitigate AD progression by dual blockade of KCs pyroptosis through inhibition of both the SLC31A1/α-KG/H3K9me3/GSDMA and STAT3/GSDMA axis.

### Safety of CaP-siSlc31a1@BSP microneedle in the treatment of AD-like mouse model

**Fig. 8:**
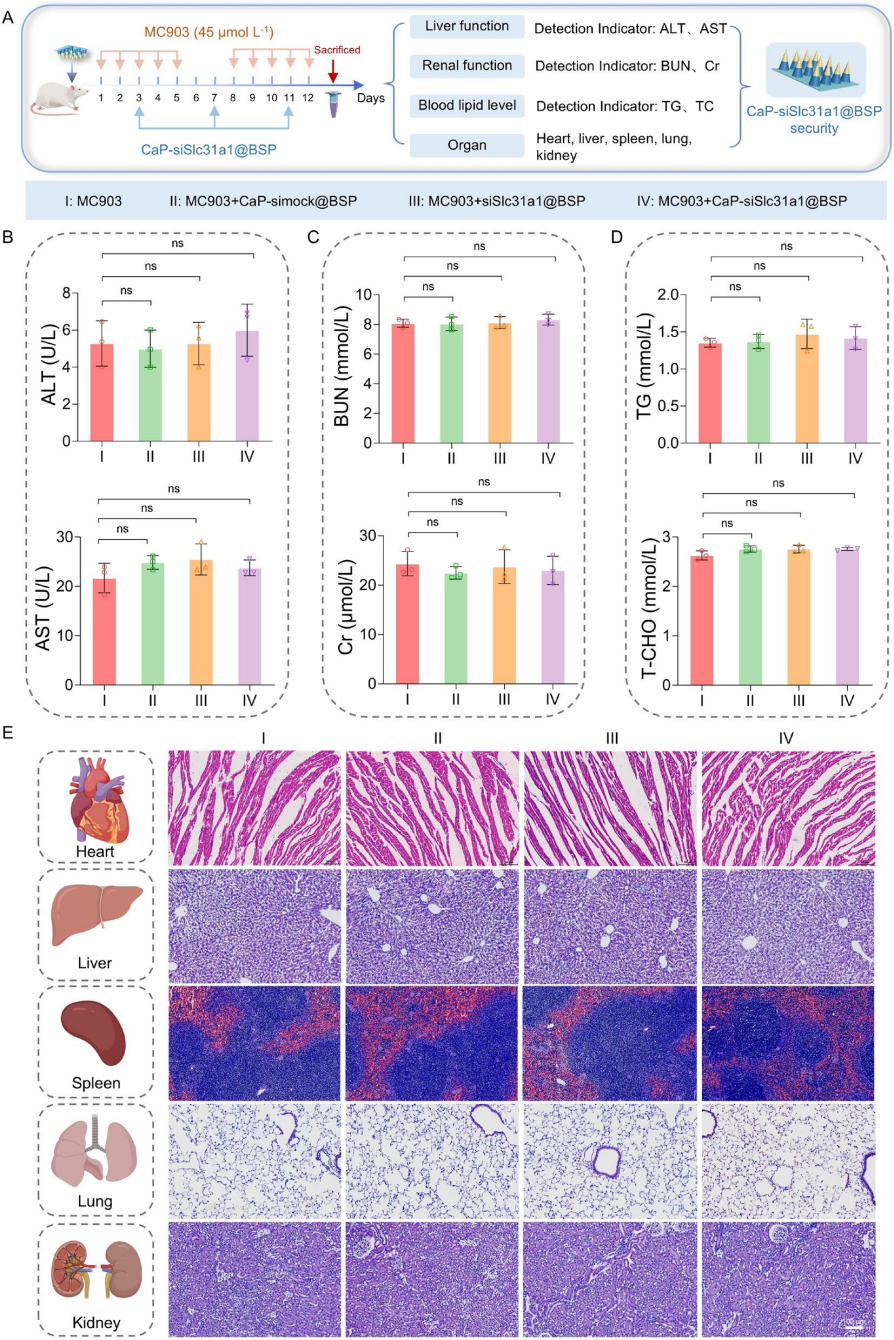
Safety of CaP-siSlc31a1@BSP microneedle for the treatment of AD. (**A**) Safety testing scheme of CaP-siSlc31a1@BSP for the treatment of AD. (**B**) Effect of CaP-siSlc31a1@BSP on liver function in AD-like mice. (**C**) Effect of CaP-siSlc31a1@BSP on renal function in AD-like mice. (**D**) Effect of CaP-siSlc31a1@BSP on blood lipid level in AD-like mice. (**E**) Histological evaluation of organ tissue (H&E staining), including heart, liver, spleen, lung, kidney, in AD-like mouse models after CaP-siSlc31a1@BSP treatment. The codes denote the following: Ⅰ, MC903-induced mice; Ⅱ, MC903-induced mice treated with CaP-simock@BSP; Ⅲ, MC903-induced mice treated with siSlc31a1@BSP; Ⅳ, MC903-induced mice treated with CaP-siSlc31a1@BSP. Data represent SD ± mean. ns, not significant; *p < 0.05; **p < 0.01; ***p < 0.001; ****p < 0.0001

Evaluating safety is paramount in the development of any novel drug or therapeutic approach. Particularly in animal models, ensuring treatments do not induce damage to vital organs such as the liver and kidneys constitutes a core component of preclinical research. As a promising therapeutic agent, safety assessment of CaP-siSlc31a1@BSP not only facilitates understanding of its impacts on physiological functions but also provides critical scientific evidence for subsequent clinical translation. Therefore, we systematically evaluated the safety profile of CaP-siSlc31a1@BSP, including assessments of the structure and function of key organs such as the liver and kidneys (Fig. 8A). Specifically, liver function was characterized by measuring serum alanine aminotransferase (ALT) and aspartate aminotransferase (AST) levels in mice (Fig. 8B); renal function was evaluated via blood urea nitrogen (BUN) and creatinine (Cr) detection (Fig. 8C); and blood lipid levels were assessed by measuring triglycerides (TG) and total cholesterol (TC) (Fig. 8D). No significant differences were observed in these biochemical parameters between the CaP-siSlc31a1@BSP group and other groups. The staining results of H&E further revealed no obvious structural abnormalities in the heart, liver, spleen, lung, or kidneys across groups (Fig. 8E), confirming that CaP-siSlc31a1@BSP does not induce pathological changes in the internal organs of mice. Collectively, these findings demonstrate that CaP-siSlc31a1@BSP exhibits no overt organ toxicity.

## Conclusion

In summary, we identify SLC31A1 as a critical mediator linking copper-induced cuproptosis to KCs pyroptosis in the pathogenesis of AD. By designing CaP-siSlc31a1@BSP microneedle, we achieved localized and efficient siRNA delivery while simultaneously exploiting the intrinsic anti-inflammatory properties of BSP. This dual-functional platform effectively silenced SLC31A1, suppressed pyroptosis-related inflammation, and alleviated AD symptoms in vitro and in vivo. Mechanistically, CaP-siSlc31a1@BSP disrupted both the SLC31A1/α-KG/H3K9me3/GSDMA and STAT3/GSDMA pathways, providing dual blockade of KCs pyroptosis. Beyond establishing SLC31A1 as a promising therapeutic target, our findings highlight CaP-siSlc31a1@BSP microneedle as a synergistic drug-gene transdermal delivery system with significant potential for the precise and translational treatment of chronic inflammatory skin disorders.

## Experimental section

### Materials

Calcipotriol (MC903) was purchased from MedChemExpress Co., Ltd (USA). Copper Assay Kit, GSH Assay Kit, Urea Assay Kit, Creatinine (Cr) Assay kit, Alanine aminotransferase Assay Kit, Aspartate aminotransferase Assay Kit, Triglyceride assay kit and Total cholesterol Assay Kit were bought from Jiancheng Bioengineering Institute (Nanjing, China). 2′,7′-Dichlorofluorescin (DCFH-DA) was provided by Merck Co., Ltd (Germany). The siSlc31a1 was obtained from RiboBio Co., Ltd (GCATGATGATGATGCCTAT, Guangzhou, China). Bovine serum albumin (BSA) was obtained from Servicebio Co., Ltd (Wuhan, China). Calcium chloride dihydrate (CaCl_2_⋅2H_2_O) was purchased from Aladdin Co., Ltd (Shanghai, China). Hoechst 33,342, Bicinchoninic Acid Assay Kit and Trypsin–EDTA Solution were obtained from Beyotime Co., Ltd (Shanghai, China). LDH-Glo™ Cytotoxicity Assay Kit was provided by Promega Co., Ltd (USA). Dispase II, α-KG, collagenase type IV and DNase I were purchased from Sigma-Aldrich Co., Ltd (USA). Keratinocyte Growth Medium 2 was purchased by ScienCell Co., Ltd (USA). Histone Extraction Kit was obtained from Thermo Fisher Scientific Co., Ltd (USA). *Bletilla striata* polysaccharide (BSP) was obtained from ZelangBiotech Co., Ltd (Xian, China). Hyaluronic acid (HA, Mw 10 kDa) was provided by MeilunBio Co., Ltd (Dalian, China). α-KG Assay Kit was obtained from Solarbio Co., Ltd (Beijing, China). SimpleChIP® Enzymatic Chromatin IP Kit (Magnetic Beads), anti-H3K9me3, anti-H3, and anti-P-STAT3 were purchased by CST Co., Ltd (US). Anti-GSDMA were provided by Abcam Co., PLC (USA). Anti-STAT3 was purchased by Santa Cruz Biotechnology Co., Inc (USA). Anti-DLAT, anti-FDX1, anti-LIAS, anti-SLC31A1, anti-β-actin, anti-PCNA, and anti-P63 were obtained from Proteintech Group Co., Inc (Wuhan, China). All primers were obtained from Sangon Biotech Co., Ltd (Shanghai, China). All antibodies for flow cytometry, as well as interferon-γ (IFNγ) and tumor necrosis factor-α (TNFα), were bought from BioLegend Co., Ltd (USA).

### Animal model

Healthy female BALB/C mice (6 to 8 weeks old) were purchased from the Department of laboratory animals of Central South University (Changsha, China). All animal studies were approved by the Experimental Animal Ethics Committee of Central South University (Approval Number: 202507123). To establish the AD-like mouse model, 25 μL MC903 (45μmol L^−1^) was applied to the right ear of each mouse for 5 days, followed by a 2-day rest, then for another 5 days. For the mouse experiment induced by α-KG, the mice were given intragastric administration of α-KG from days 7 to 12.

### Determination of copper (Cu) levels and GSH levels

Cu levels were determined using a copper Assay Kit according to the manufacturer’s instructions. In short, skin tissue was homogenized in ice-cold PBS, and the homogenate was reacted with ascorbate and 3,5-dibromo-PAESA. The Cu concentration was then quantified by recording the absorbance of the formed blue complex at 600 nm.

For the determination of GSH levels, we used the GSH Assay Kit. Briefly, Skin specimens were accurately weighed and homogenized in ice-cold saline (1:9 w/v). After centrifugation, the supernatant was collected and assayed according to the GSH Assay Kit instructions. GSH levels were determined at 405 nm with a microplate reader.

### Tissue ROS detection

The 50 to 100 mg of fresh tissue samples were added to 1mL of homogenization buffer, and homogenized thoroughly using a homogenizer, as following centrifuged at 100 × g for 3 min at 4 °C. 10 μL of the homogenate supernatant was added with DCFH-DA probe, and incubated at 37°C in the dark for 30 min. The fluorescence intensity was measured in a fluorescent microplate reader (BioTek, USA). 50 μL of the supernatant homogenate from each sample was used for protein quantification. Tissue ROS intensity was expressed as fluorescence intensity (RFU)/ protein concentration (mg).

### Quantitative real-time polymerase chain reaction (qRT-PCR)

Total RNA was isolated from cells or tissues using MagZol reagent (Magen), and cDNA was synthesized via reverse transcription using a HiScript Q RT Kit (Yeasen). Two-step real-time RT-PCR was performed using SYBR Green Reagent (Bimake). The primer sequences are provided in Table S1. The results were analyzed using the 2^−ΔΔCt^ method, and the data are presented as the ratio relative to the control gene β-actin.

### Western blot

The protein samples were obtained from cells or tissues with RIPA buffer, and measured by the Bicinchoninic Acid Assay Kit. Proteins were separated by 10% sodium dodecyl sulfate polyacrylamide gel electrophoresis (SDS-PAGE) and transferred to PVDF membranes. After blocked, the PVDF membranes were incubated with specific antibody for FDX1, LIAS, DLAT, GSDMA, H3K9me3, H3, P-STAT3, and β-actin overnight. Followed by mixed with second antibody, the results were visualized by ChemiDoc XRS + System (BIO-RAD, USA).

### Histopathological staining

Paraffin-embedded sections of healthy and AD human skin were obtained with approval from the Medical Ethics Committee of Xiangya Hospital and informed consent from all subjects (Approval Number: 202308636). Paraffin-embedded sections of mice skin samples were obtained from the treated AD-like mouse model.

After heated at 60 °C for 4 h, the paraffin sections were soaked through a graded series of dimethylbenzene and ethanol sequentially. Then the sections were heated with citrate antigen retrieval solution at 100 °C for 21 min, blocked with 10% goat serum and incubated with primary antibodies (SLC31A1, PCNA, P63) overnight, followed by incubation with secondary antibodies. For immunohistochemistry, signals were detected using an enzyme-labeled secondary antibody and DAB reagent, and slides were counterstained using hematoxylin. For immunofluorescence staining, the sections were observed using the fluorescence microscope. H&E staining was performed on paraffin-embedded sections using a commercial H&E staining kit according to the manufacturer’s instructions.

### Preparation and characterization of CaP-siSlc31a1

BSA (300 mg) was dissolved in 30 mL DMEM medium, followed by 60 nmol siSlc31a1 diluted with 600 μL DEPC treated water was added. Subsequently, 300 μL CaCl_2_ (1 mol L^−1^) was added to the aforementioned solution. The mixture was incubated at 37 °C for 24 h. Then, the CaP-siSlc31a1 was isolated by centrifugation at 12,000 rpm for 15 min. The loading capacity of siSlc31a1 was assessed by using a fluorescence microplate reader (Synergy2, Biotek, USA). For morphological characterization and elemental analysis, transmission electron microscopy (TEM, Tecnai G2 F20, FEI, USA) was used. Additionally, the hydrodynamic diameters of nanoparticles were determined by using a Malvern Zetasizer (Nano ZS90, UK).

### Isolation and culture of mouse primary KCs

Mouse primary KCs were isolated from newborn mice. The isolated skin was digested in either 1 mL of DPBS containing 2 mg mL^−1^ dispase II at 4 °C for 16 to 20 h. Then, the epidermis was torn off using tweezers. Next, the epidermis was digested in 1 mL of 0.25% Trypsin–EDTA Solution at 37 °C for 5 to 10 min. After this, Trypsin/EDTA activity was stopped with the addition of 2 mL of DMEM containing 10% fetal bovine serum. The KCs were obtained by centrifugation (1000 rpm at room temperature for 3 min) and cultured using Keratinocyte Growth Medium 2. The cells in the plate were cultured as normal primary KCs. All cells were cultured in Keratinocyte Growth Medium 2 in a humidified atmosphere containing 5% CO_2_ at 37 °C. Experiments on primary cells were conducted 24 h after isolation. Cell morphology was observed under a microscope.

### Cellular uptake and lysosomal escape

For the cellular uptake study, mouse primary KCs were seeded into a 6-well plate and allowed to adhere overnight at 37 °C with 5% CO_2_. Following this, the cells were incubated with FAM-labeled nanoparticles for 24 h. Post-incubation, the cells were washed and collected. The obtained cells were detected by flow cytometry to measure fluorescence intensity. For the lysosomal escape study, cells were seeded into confocal dishes and cultured overnight under the same conditions as mentioned above. The following day, the cells were incubated with FAM-labeled CaP-siSlc31a1 for 0.5, 1, 2, 4, and 6 h. At predetermined time, the cells were stained with Lyso-Tracker Red and Hoechst 33,342. Finally, the treated cells were imaged using a confocal laser scanning microscopy (CLSM) system (ZEISS, Germany).

### Determination of LDH-Glo™ cytotoxicity assays

Cell viability and death were assessed using the LDH-Glo™ Cytotoxicity Assay Kit per the manufacturer’s instructions. KCs were seeded in a 96-well plate and cultured for 24 h at 37 °C prior to treatment. To determine maximum LDH release, cells were lysed by adding 2 µL of TritonX-100 per 100 µL of medium and incubated for 15 min. For measuring LDH released from non-viable cells, 10 µL of culture medium was removed and diluted with 490 µL of LDH storage buffer. Then, 50 µL of supernatant from all test and control wells was transferred to a fresh 96-well flat-bottom plate. Subsequently, 50 µL of LDH Detection Reagent was added and incubated at room temperature in the dark for 30 min. Absorbance at 490 nm was recorded using a BioTek Synergy Neo2 plate reader. 50 µL of culture medium was measured to establish the baseline LDH activity. The formula for computing percent cytotoxicity: Percent cytotoxicity = 100 × (Experimental LDH release–Baseline LDH [OD_490_]/maximum LDH release–Baseline LDH [OD_490_]).

### Histone extraction

Histones were extracted using a Histone Extraction Kit. Cells were harvested, counted, and centrifuged at 200 × g for 5 min. The pellet was washed twice with DPBS, followed by another centrifugation step at 200 × g for 5 min, and the supernatant was discarded. The pellet was resuspended in 100 µL of ice-cold extraction buffer per 1 × 10⁶ cells and incubated for 2 h at 4 °C on an end-over-end rotator. After incubation, the sample was centrifuged at 20,800 × g for 10 min, and the supernatant was collected.

### Chromatin immunoprecipitation (ChIP) assay

The ChIP assay was performed using a SimpleChIP® Enzymatic Chromatin IP Kit (Magnetic Beads) following the manufacturer’s protocol. Cells were cross-linked, lysed, and sonicated. The antibodies for the ChIP assay were H3K9me3, STAT3 and IgG. Chromatin was immunoprecipitated with protein-DNA complexes isolated from protein A/G beads. The binding sites of H3K9me3 and GSDMA promoter were predicted using the Cistrome Data Browser database (http://cistrome.org/db/). Using JASPAR database (https://jaspar.elixir.no/) predict STAT3 and GSDMA promoter of binding sites. Purified DNA was analyzed by qRT-PCR using primers for the GSDMA promoter. The qRT-PCR primers of the GSDMA promoter used in the ChIP assay are listed in Table S2 and S3.

### Fabrication and characterization of microneedles

The obtained CaP-siSlc31a1 suspension was mixed with 5% (w/v) BSP solution, and the mixture was transferred into molds and centrifuged at 4500 rpm for 30 min. Then, the samples were allowed to dry completely at 30 °C for 4 h. Subsequently, 50% (w/v) HA solution was added. After dried for another 24 h, the samples were demolded to obtain CaP-siSlc31a1@BSP.

The morphological characteristics of CaP-siSlc31a1@BSP was examined by optical microscopy (ZEISS, Germany) and scanning electron microscopy (SEM, JSM-6330 F, JEOL, Japan). To visualize the distribution of nanoparticles within the CaP-siSlc31a1@BSP, fluorescence microscopy (Leica, Germany) was employed. The mechanical strength of CaP-siSlc31a1@BSP was estimated using an electronic universal testing machine (CMT6103, China).

### Skin insertion capability and dissolvability

To assess the insertion ability of CaP-siSlc31a1@BSP, the microneedles were applied to the ventral surface of the ears on mice. The treated ear skin tissues were harvested and processed for histological examination. Microneedles were applied to the mice ear skin and examined using the optical microscope to assess dissolvability of CaP-siSlc31a1@BSP at predetermined time intervals.

### CaP-siSlc31a1 accumulation in vivo

To determine the distribution of CaP-siSlc31a1 following microneedle delivery, the microneedles were applied to the ear skin in mice for 5 min. After administration for 2, 24, 48, 72 and 96 h, the treated mice were put on an IVIS Spectrum (Perklin Elmer, Germany) for imaging.

### In vivo treatment efficiency

For the MC903-induced AD-like mouse experiment treated with MNs, the mice were randomly assigned to five groups (n = 5 per group) as follows: I) Con, II) MC903, III) MC903 + CaP-simock@BSP, IV) MC903 + siSlc31a1@BSP, V) MC903 + CaP-siSlc31a1@BSP. Different types of MNs were applied locally to each mouse on days 3, 7 and 11. The MNs were firmly pressed for 60 s to penetrate the stratum corneum and epidermis, and then gently pressed for another 30 s to allow the MNs to absorb the liquid in the skin. The skin lesions and body weight of the mice were observed and recorded daily. The thickness of skin folds was measured using a vernier caliper (Mitutoyo, Japan). During the treatment, the AD severity score was recorded as the sum of scores graded as 0 (no symptoms), 1 (mild), 2 (moderate), or 3 (severe) for each of the four measured symptoms (erythema/hemorrhage, excoriation/erosion, edema, and scarring/dryness). The scratching behavior of AD-like mice was recorded by video, and the videos were analyzed. Before observing scratching, the AD-like mice were acclimated in the observation room for at least 30 min. Itch behavior was assessed by quantifying the number of scratching bouts over a 30-min observation period. After 12 days, the mice were sacrificed to obtain skin tissue. In the histological analysis, the skin was fixed in 4% paraformaldehyde and stained with H&E for microscopic observation.

### Flow cytometry

The ear skin tissue of mice after modeling was collected (about 3*3 mm/ sample). Skin samples were cut into small pieces and digested in 3 mL of DMEM containing 2 mg mL^−1^ collagenase type IV and 100 μg mL^−1^ DNase I. The enzyme activity was terminated by using 10% fetal bovine serum DMEM medium, and the resulting single-cell suspension was obtained after filtration through cell strainer. To assess the CD4^+^ T cells infiltration, antibodies used to stain single-cell suspensions were as follows: PerCP/Cyanine5.5 anti-mouse CD4 Antibody, APC/Cyanine7 anti-mouse CD45 Antibody, PE anti-mouse IL-4 Antibody and APC anti-mouse IL-17A Antibody. The cells were then acquired using a BD LSR II flow cytometer (BD Biosciences) and analyzed using FlowJo software (TreeStar).

### Targeted metabolomics

Cell pellets were extracted with 1 mL ice-cold methanol/acetonitrile/water (2:2:1, v/v) by 1 h sonication on ice, held at -20 °C for 1 h, and centrifuged (16,000 g, 4 °C, 20 min). Supernatants were dried, reconstituted in 50% acetonitrile, filtered (0.22 μm), and stored at -80 °C. A pooled Quality control sample was injected at regular intervals. Metabolites were analyzed on a Shimadzu Nexera X2 LC-30AD coupled to a 5500 QTRAP MS using an ACQUITY UPLC HSS T3 column (40 °C, 0.2 mL min^−1^) and a 0.1% Formic acid-acetonitrile gradient; detection was performed in positive/negative electrospray ionization multiple-reaction monitoring mode and peak areas were extracted with MultiQuant 3.0.2.

### KEGG enrichment analysis

To identify the perturbed biological pathways, the differential metabolite data were performed KEGG pathway analysis using KEGG database (http://kegg.jp). KEGG enrichment analyses were carried out with the Fisher’s exact test, and FDR correction for multiple testing was also performed.

### Determination of α-KG levels assays

According to the α-KG Assay Kit protocol, an equal volume of Extraction Solution 1 was added to the homogenate. This mixture was kept on ice and centrifuged (12,000 rpm, 10 min, 4 °C). Subsequently, 0.8 mL of the supernatant was gently mixed with 0.15 mL of Extraction Solution 2 until no bubbles remained. The sample was centrifuged again under the same conditions, and the resulting supernatant was used for subsequent assays as specified by the manufacturer.

### Statistical analysis

All statistical analyses were performed using GraphPad Prism 9 (GraphPad Software, San Diego, CA, USA). When the sample was not normally distributed, the statistical significance between the values was determined using either a 2-tailed unpaired Student’s t-test or one-way ANOVA with Dunnett’s post hoc test. The correlation between the measured variables was tested by Spearman rank correlation analysis. All data are presented as the mean ± SD. ns, not significant; *p < 0.05; **p < 0.01; ***p < 0.001; ****p < 0.0001.

## Supplementary Information

Additional file 1.

Additional file 2.

## Data Availability

All data generated or analyzed during this study are included in article and/or its supplementary material files. Further enquiries can be directed the corresponding author.

## Abbreviations

AD: Atopic dermatitis
α-KG: α-Ketoglutarate
ALT: Alanine aminotransferase
AST: Aspartate aminotransferase
BSA: Bovine serum albumin
BSP: *Bletilla striata* polysaccharide
BUN: Blood urea nitrogen
CaP: Calcium phosphate
CaP-siSlc31a1: SiSlc31a1-loaded CaP nanoparticles
CaP-siSlc31a1@BSP: Calcium phosphate nanoparticles delivering Slc31a1 siRNA and embedded within *Bletilla striata* polysaccharide microneedles
ChIP: Chromatin immunoprecipitation
CLSM: Confocal laser scanning microscopy
Cr: Creatinine
DAPI: 4’,6-Diamidino-2-phenylindole
DLS: Dynamic light scattering
DLAT: Dihydrolipoamide S-acetyltransferase
DMEM: Dulbecco’s modified Eagle’s medium
EASI: Elevated eczema area and severity index
FAM-siSlc31a1: FAM-labeled siSlc31a1
GSH: Glutathione
HA: Hyaluronic acid
H&E: Hematoxylin-eosin
IFNγ: Interferon-gamma
KCs: Keratinocytes
KDM5B: Lysine demethylase 5B
LDH: Lactate dehydrogenase
MC903: Calcipotriol
MN: Microneedle
PBS: Phosphate-buffered saline
PCNA: Proliferating cell nuclear antigen
P-STAT3: Phosphorylated STAT3
ROC: Receiver operating characteristic
ROS: Reactive oxygen species
qRT-PCR: Quantitative reverse transcription polymerase chain reaction
SEM: Scanning electron microscopy
siRNA: Small interfering RNA
siSlc31a1: Slc31a1 siRNA
SLC31A1: Solute carrier family 31 member 1
TC: Total cholesterol
TCA: Tricarboxylic acid
TEM: Transmission electron microscopy
TG: Triglycerides
TNFα: Tumor necrosis factor-α
