# Supplementary material for: Microneedle-mediated transdermal delivery of siRNA-loaded nanoparticles for atopic dermatitis therapy by disrupting cuproptosis-pyroptosis crosstalk
Source: J Nanobiotechnology. 2026 May 18;24:654. doi: 10.1186/s12951-026-04533-9 (PMC13362213; doi:10.1186/s12951-026-04533-9)
Supplement: Supplementary file 1 — Additional file 1. [file 12951_2026_4533_MOESM1_ESM.docx]

**Supporting information**

**Microneedle-mediated Transdermal Delivery of siRNA-loaded Nanoparticles for Atopic Dermatitis Therapy by Disrupting Cuproptosis-Pyroptosis Crosstalk**

**Authors:** Pian Yu ^1, 3, 4, 5, 6^**^#^**, Chi Fang ^1, 3, 4, 5, 6^**^#^**, Zhisheng Luo ^2^**^#^**, Lu Hao ^2^, Kaixuan Li ^1, 3, 4, 5, 6^, Rongxuan Yan ^1, 3, 4, 5, 6^, Sihui Ma ^1, 3, 4, 5, 6^, Guanming Wang ^1, 3, 4, 5, 6^, Qiaozhi Cao ^1, 3, 4, 5, 6^, Jie Dong ^8^, Xiang Chen ^1, 3, 4, 5, 6*^, Jie Li ^1, 3, 4, 5, 6*^, Peng Liu ^2, 7*^, Shuo Hu ^2, 7*^,Cong Peng ^1, 3, 4, 5, 6*^

**Affiliations:**

^1^ The Department of Dermatology, Xiangya Hospital, Central South University, Changsha, Hunan 410000, China.

^2^ Department of Nuclear Medicine, Xiangya Hospital, Central South University, No. 87 Xiangya Road, Changsha, Hunan 410008, China

^3^ Hunan Key Laboratory of Skin Cancer and Psoriasis, Hunan Engineering Research Center of Skin Health and Disease, Xiangya Hospital, Changsha, Hunan 410000, China.

^4^ Furong Labratory, Changsha, Hunan 410000, China.

^5^ National Engineering Research Center of Personalized Diagnostic and Therapeutic Technology, Changsha, Hunan 410000, China.

^6^ National Clinical Research Center for Geriatric Disease, Xiangya Hospital, Changsha, Hunan 410000, China.

^7^ Key Laboratory of Biological Nanotechnology, NHC. No. 87 Xiangya Road, Changsha Hunan 410008, China

^8^ Xiangya School of Pharmaceutical Sciences, Central South University, Changsha, Hunan 410013, China.

**# These authors contributed equally to this work.**

***Correspondence:** Cong Peng, E-mail: [pengcongxy@csu.edu.cn](mailto:pengcongxy@csu.edu.cn). Shuo Hu, E-mail: [hushuo2018@163.com](mailto:hushuo2018@163.com). Peng Liu, E-mail: [liupengpharmacy@163.com](mailto:liupengpharmacy@163.com). Jie Li, E-mail: [xylijie@csu.edu.cn](mailto:xylijie@csu.edu.cn). Xiang Chen, E-mail: chenxiangck@126.com.

**
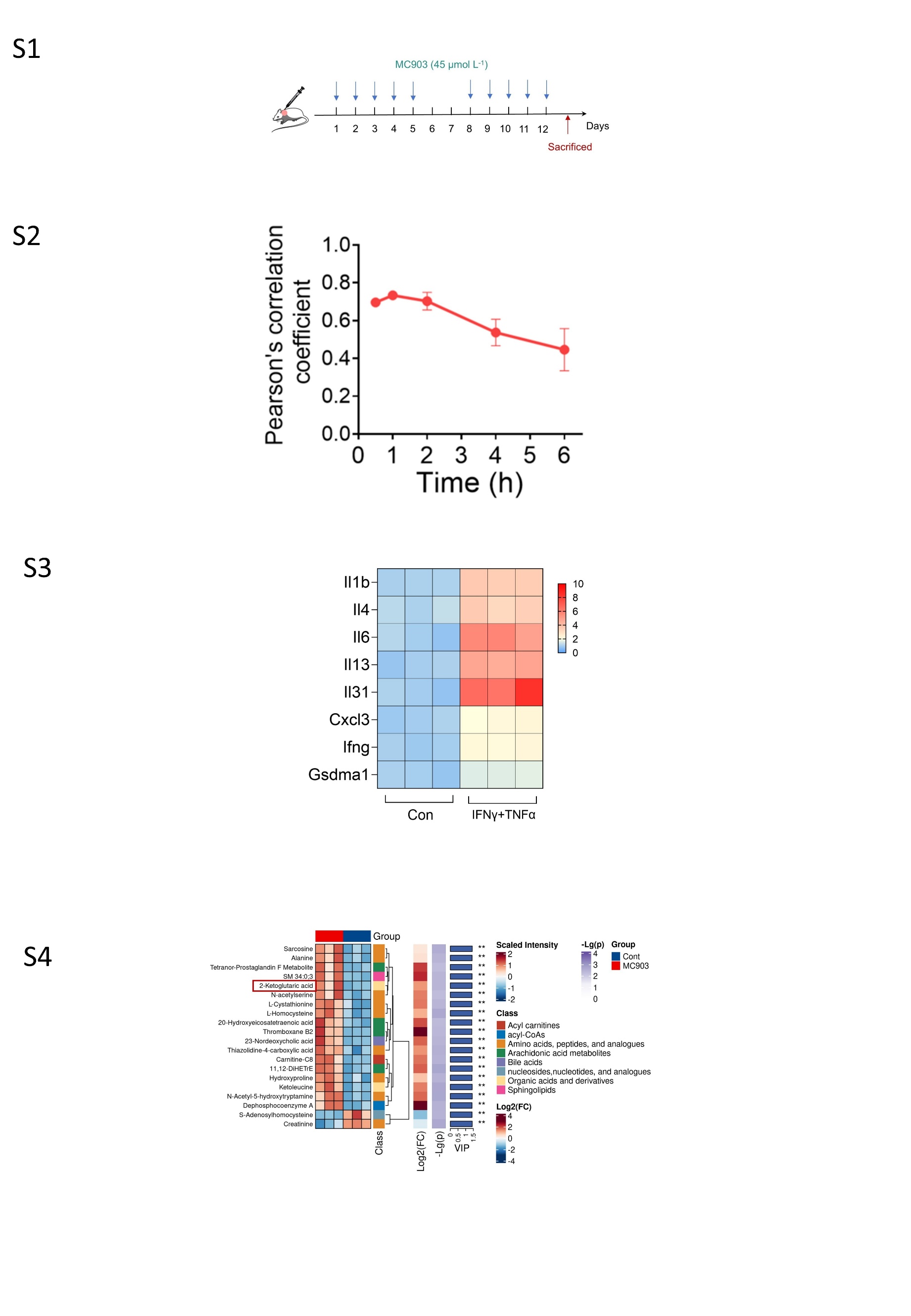
**

**Figure S1.** Schematic illustration of MC903-induced AD in mice.

**
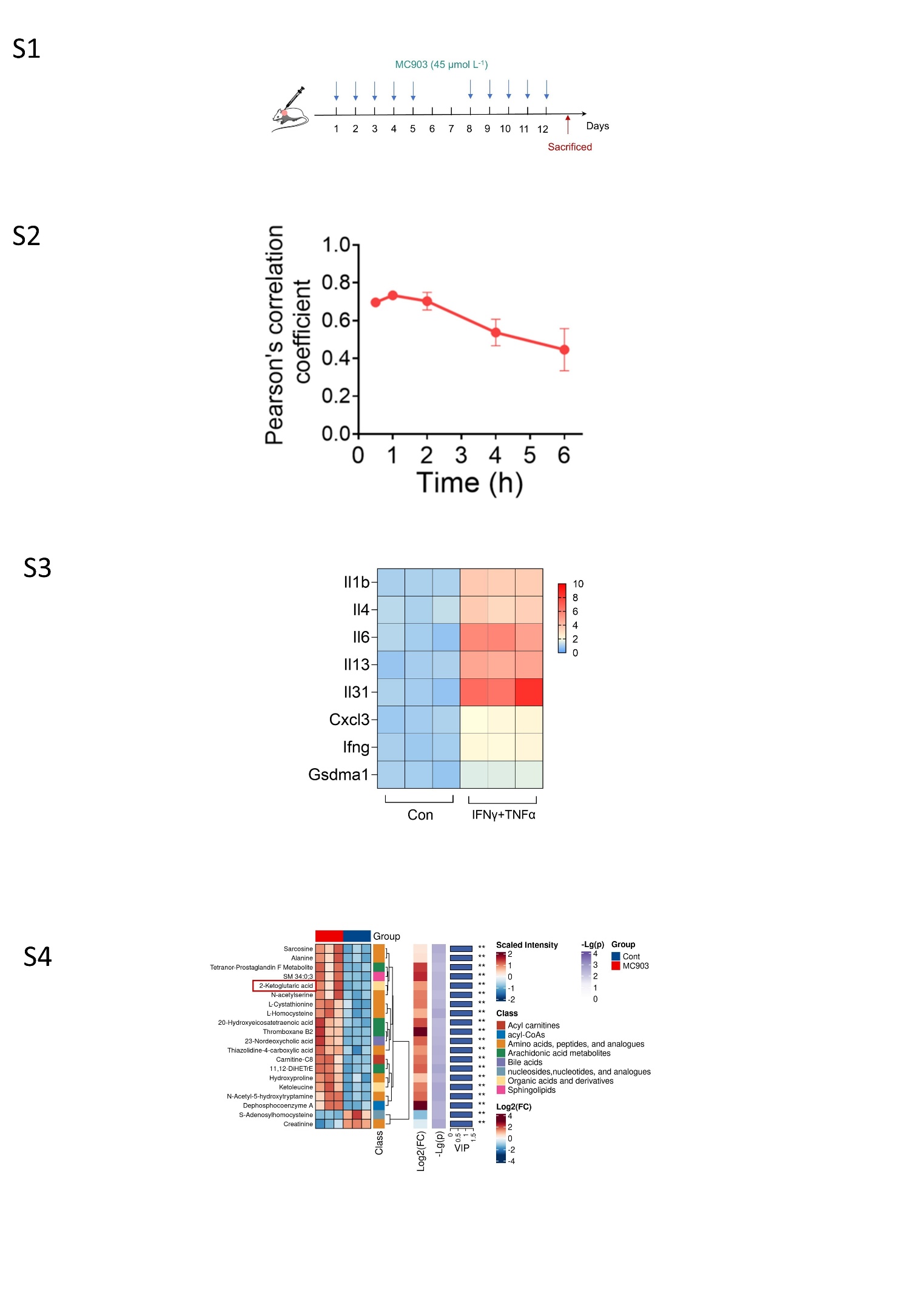
**

**Figure S2.** Evolution with time of the Pearson’s correlation coefficients between the signals from FAM-labeled CaP-siSlc31a1 and Lysotracker.

**
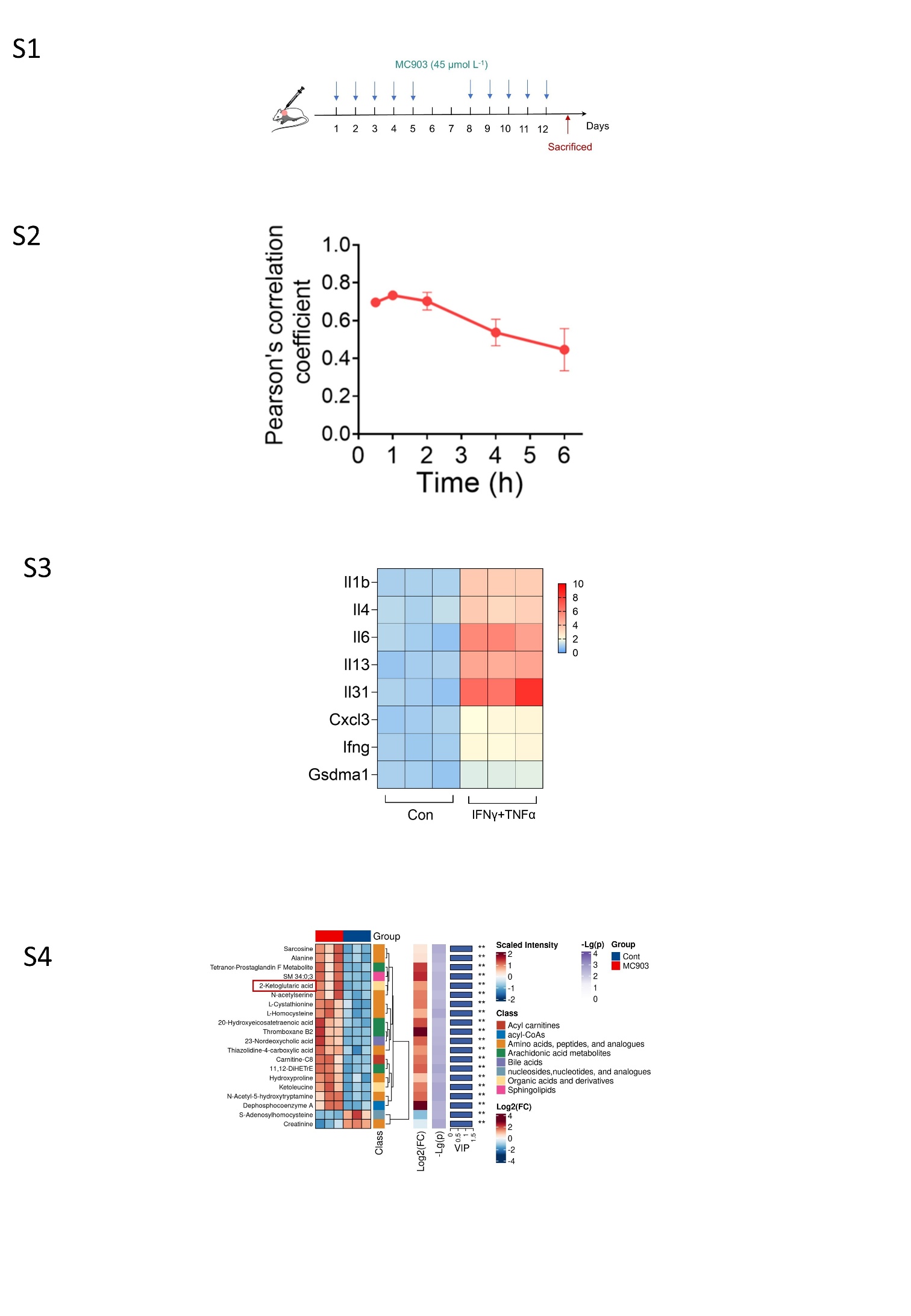
**

**Figure S3.** Relative mRNA levels of cytokines and chemokines in mouse primary KCs stimulated with IFNγ/ TNFα (10 ng mL^-1^) for 24 h in vitro (n = 3).

**
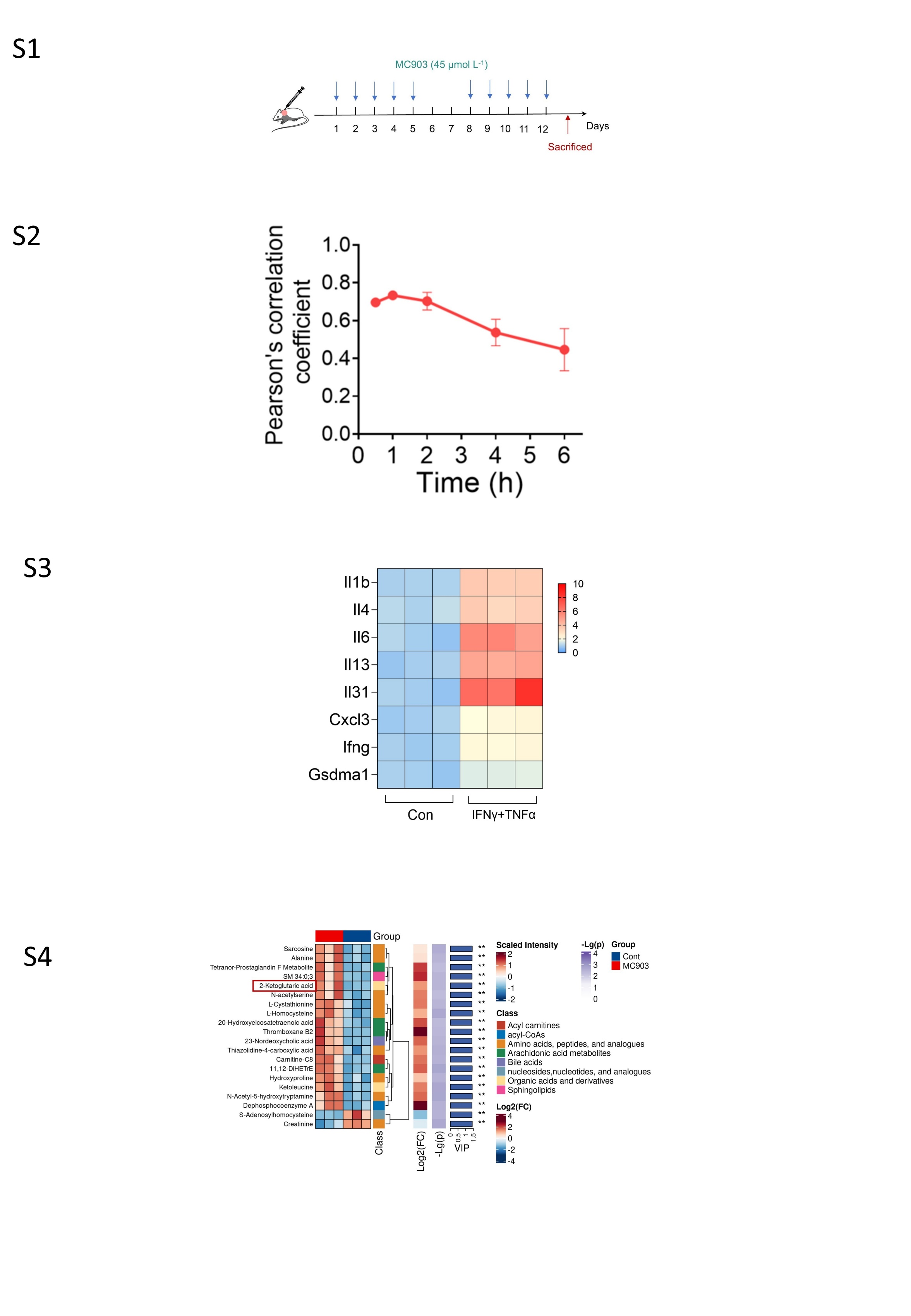
**

**Figure S4.** MC903-induced AD-like mice skin lesions were analyzed by targeted metabolomics. Abundance differences of α-KG and other metabolites (n = 3).


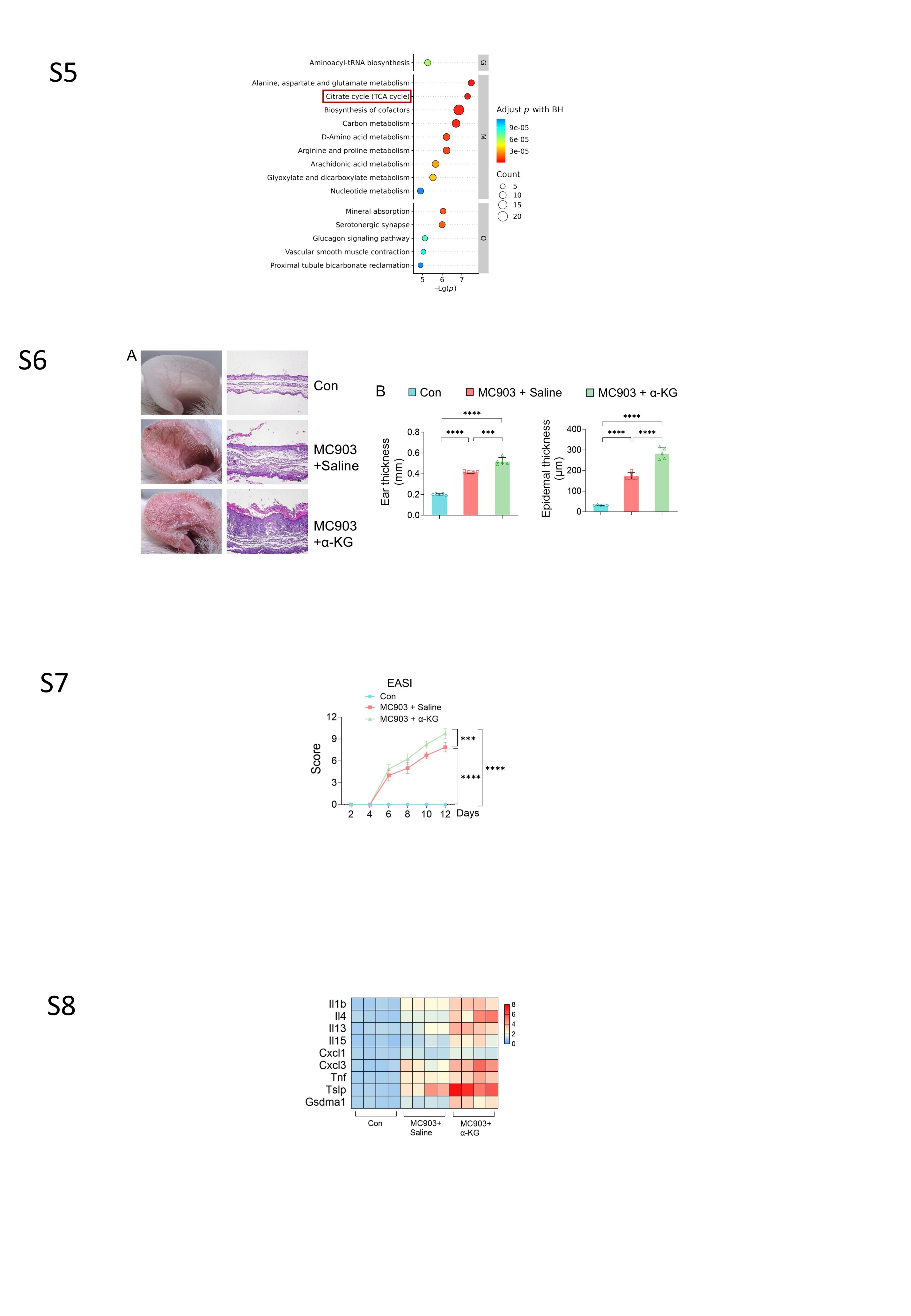


**Figure S5.** Correlation of metabolites identified in skin lesions with pathways such as the TCA cycle.


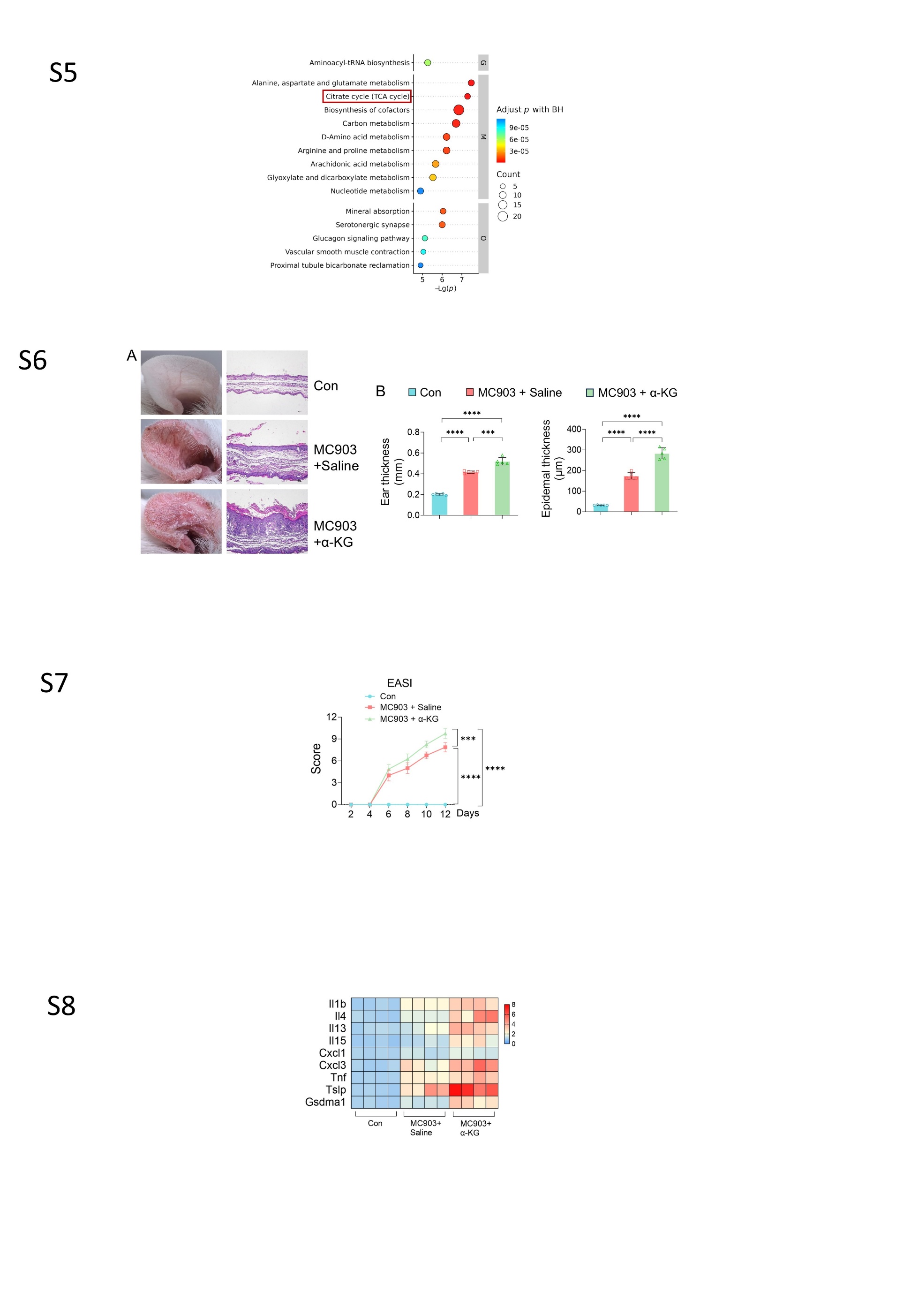


**Figure S6.** Images of MC903-induced AD-like mice treated with α-KG and macroscopic views of ear skin stained with H&E. (A) Representative images of mice and macroscopic views of ear skin stained with H&E. One representative mouse per group is shown (n = 5). Scale bar: 100 μm. (B) Statistical analysis of ear thickness and epidermal thickness.


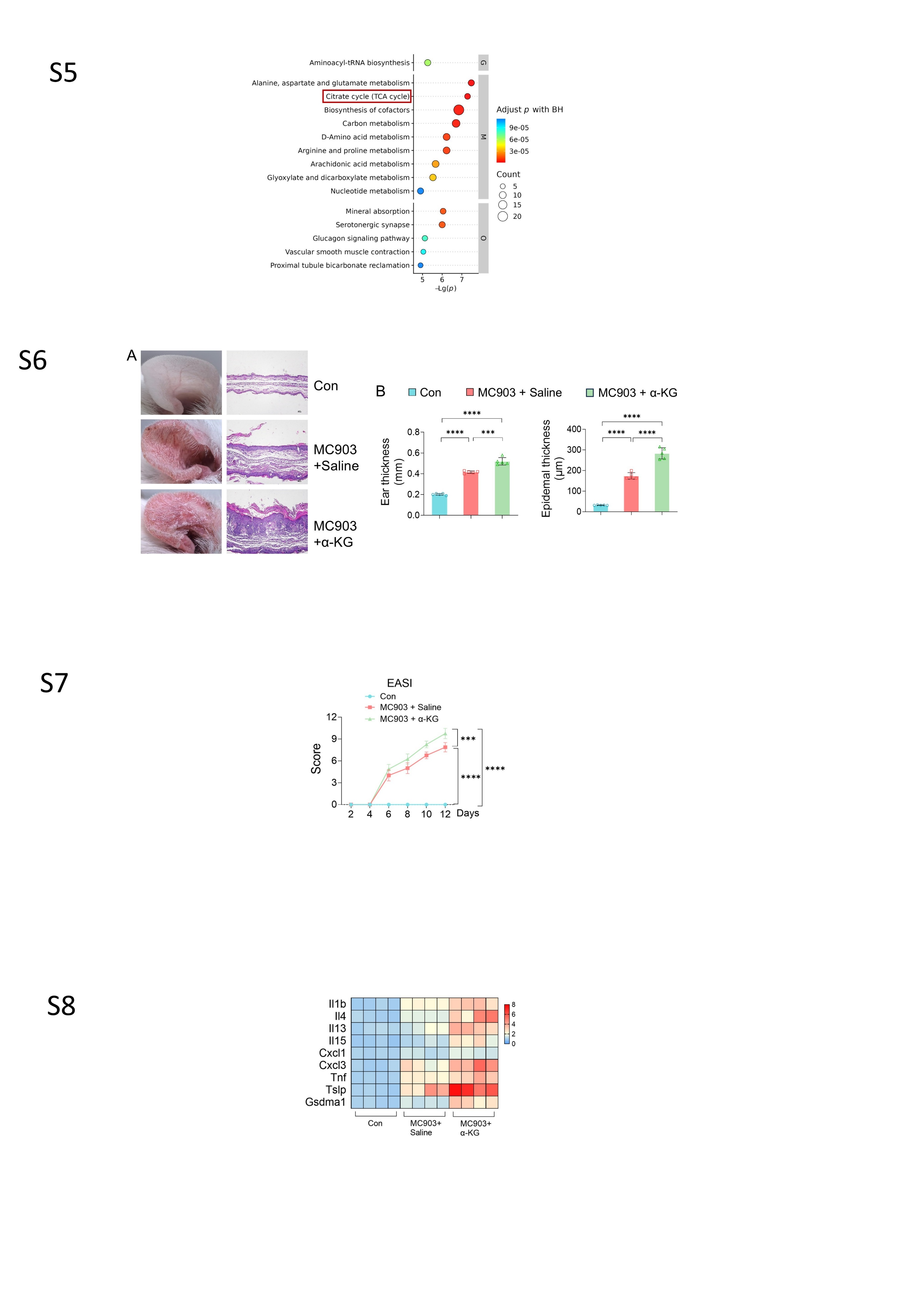


**Figure S7.** Severity scores were measured every two days for 12 days in α-KG-treated MC903-induced AD-like mice.


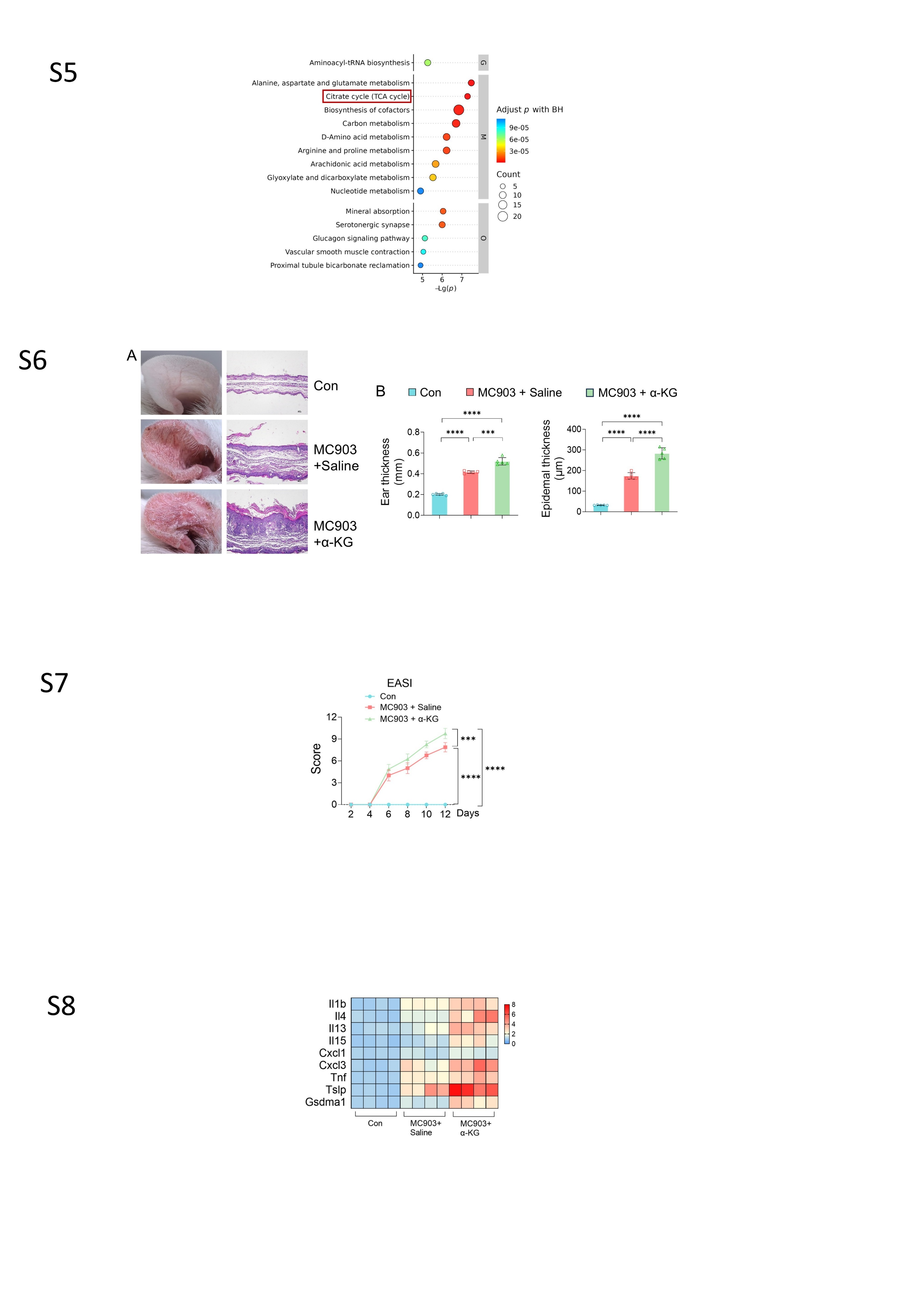


**Figure S8.** Relative mRNA levels of cytokines and chemokines in skin lesions of α-KG-treated MC903-induced AD-like mice.


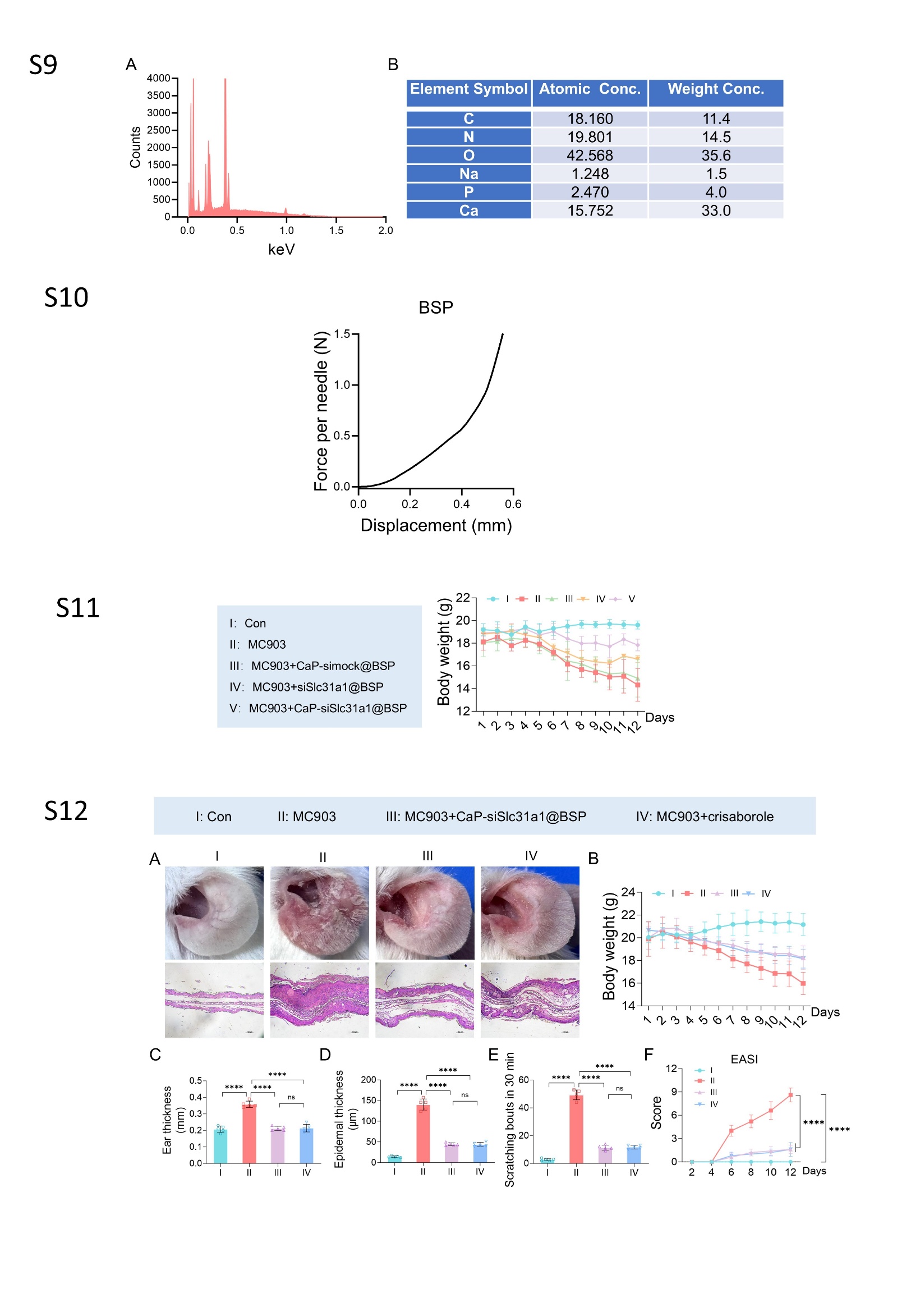


**Figure S9.** Elemental analysis of CaP-siSlc31a1@BSP tips.


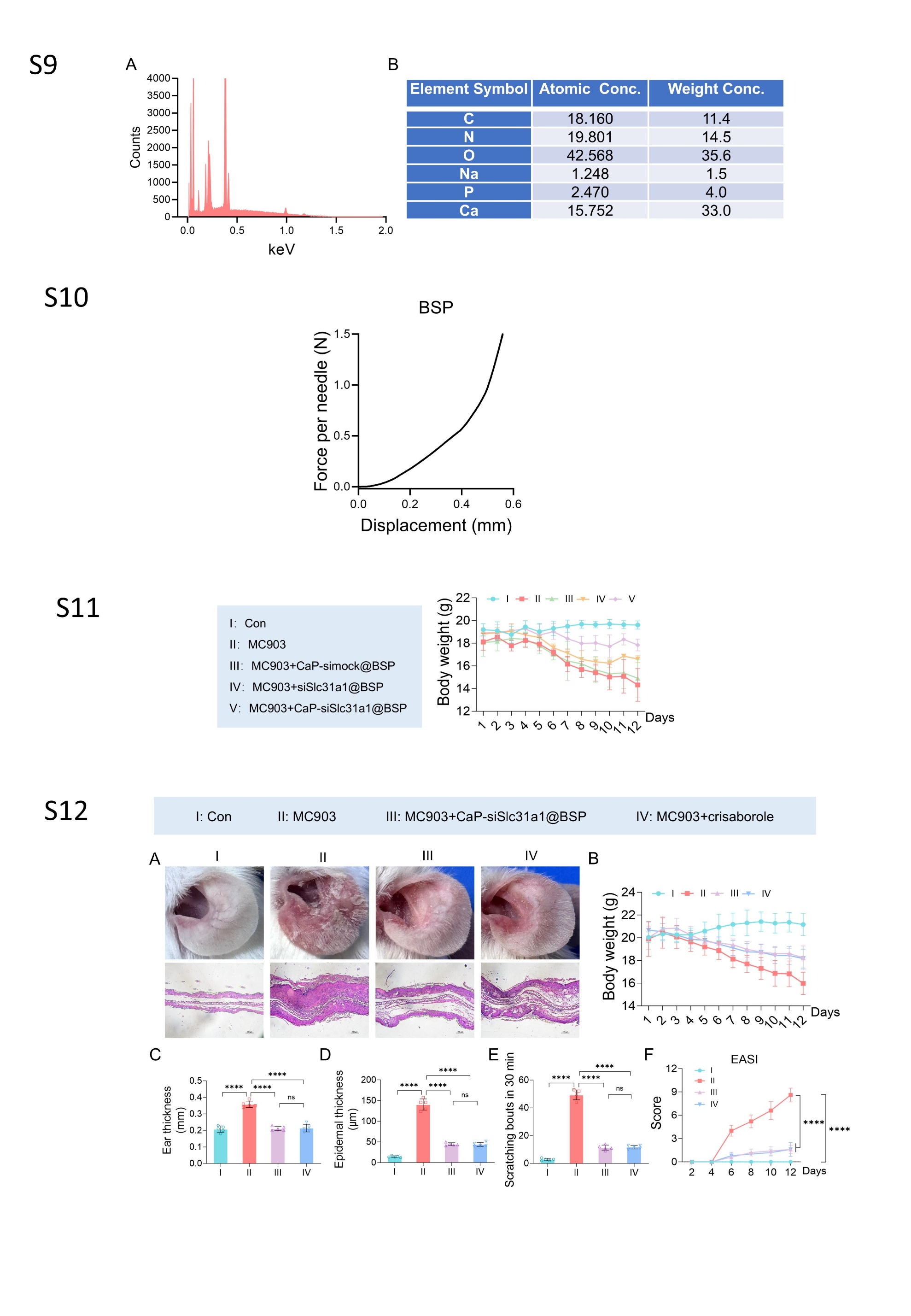


**Figure S10.** Force-displacement profiles of BSP microneedle.


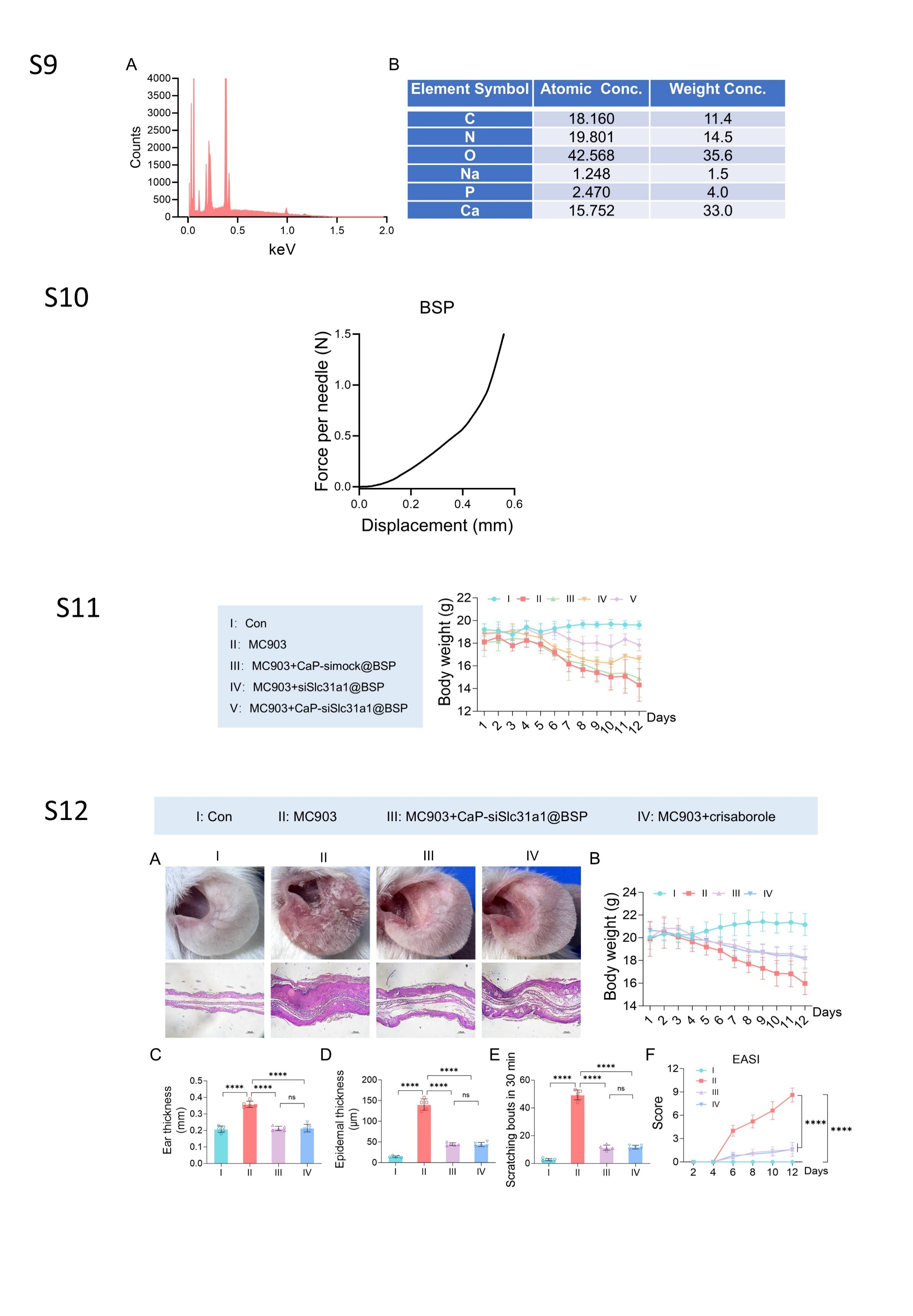


**Figure S11.** Statistical data on body weight of mice following specified treatments.


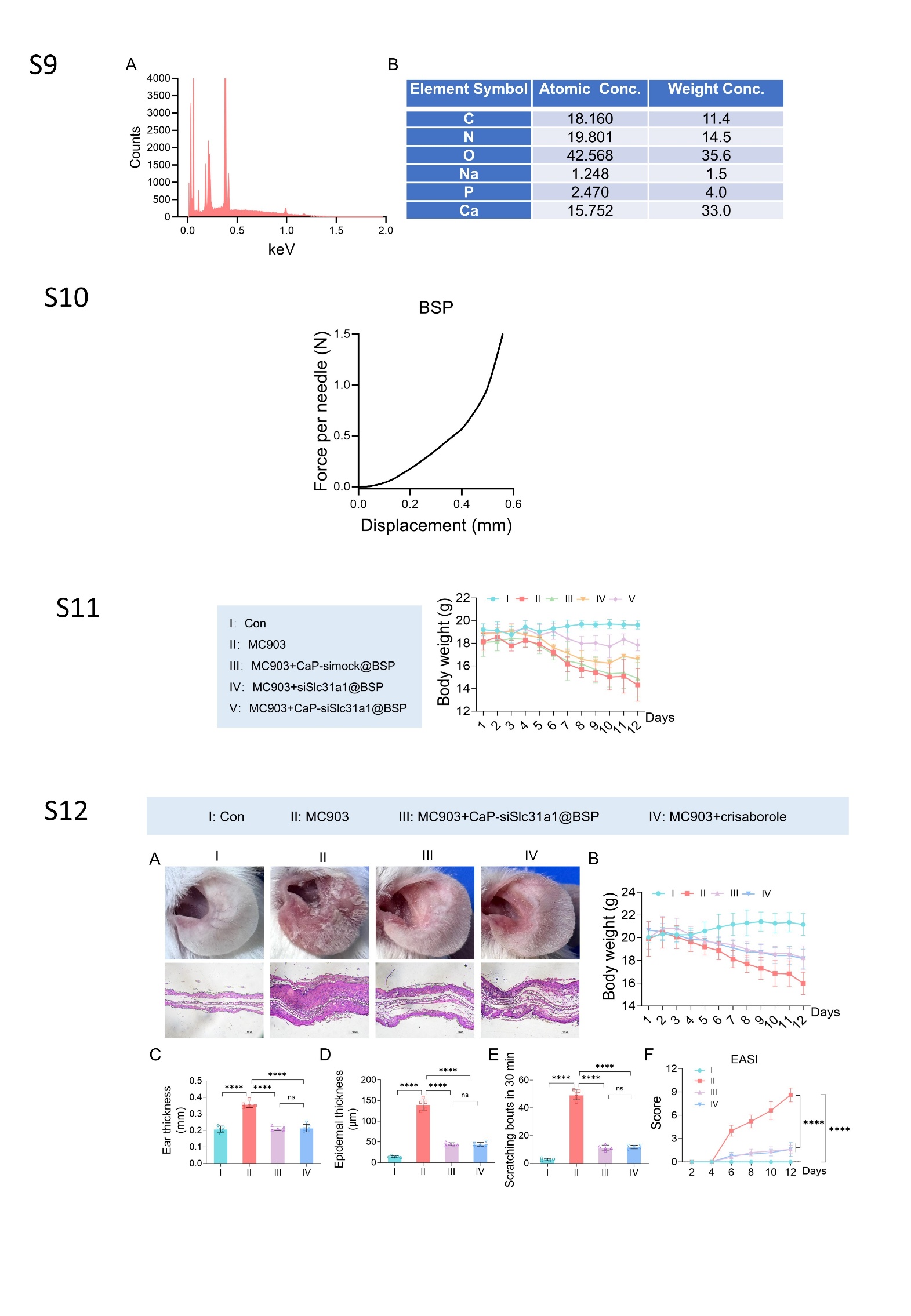


**Figure S12.** (A) Representative images of MC903-induced AD-like mice after treatment with microneedles or crisaborole, and macroscopic views of ear skin stained with H&E. One representative mouse per group is shown (n = 5). Scale bar: 100 μm. (B) Statistical data on body weight of mice following specified treatments. (C) Statistical analysis of ear thickness and (D) epidermal thickness in mice following designated treatments. (E) Scratching bouts within a 30-min period on day 12 following designated treatments. (F) Severity scores of mice, measured biweekly during the 12-day treatment period following designated interventions. The codes denote the following: Ⅰ, normal mice; Ⅱ, MC903-induced mice; Ⅲ, MC903-induced mice treated with CaP-siSlc31a1@BSP; Ⅳ, MC903-induced mice treated with crisaborole. Data represent SD ± mean. ns, not significant; *p < 0.05; **p < 0.01; ***p < 0.001; ****p < 0.0001.


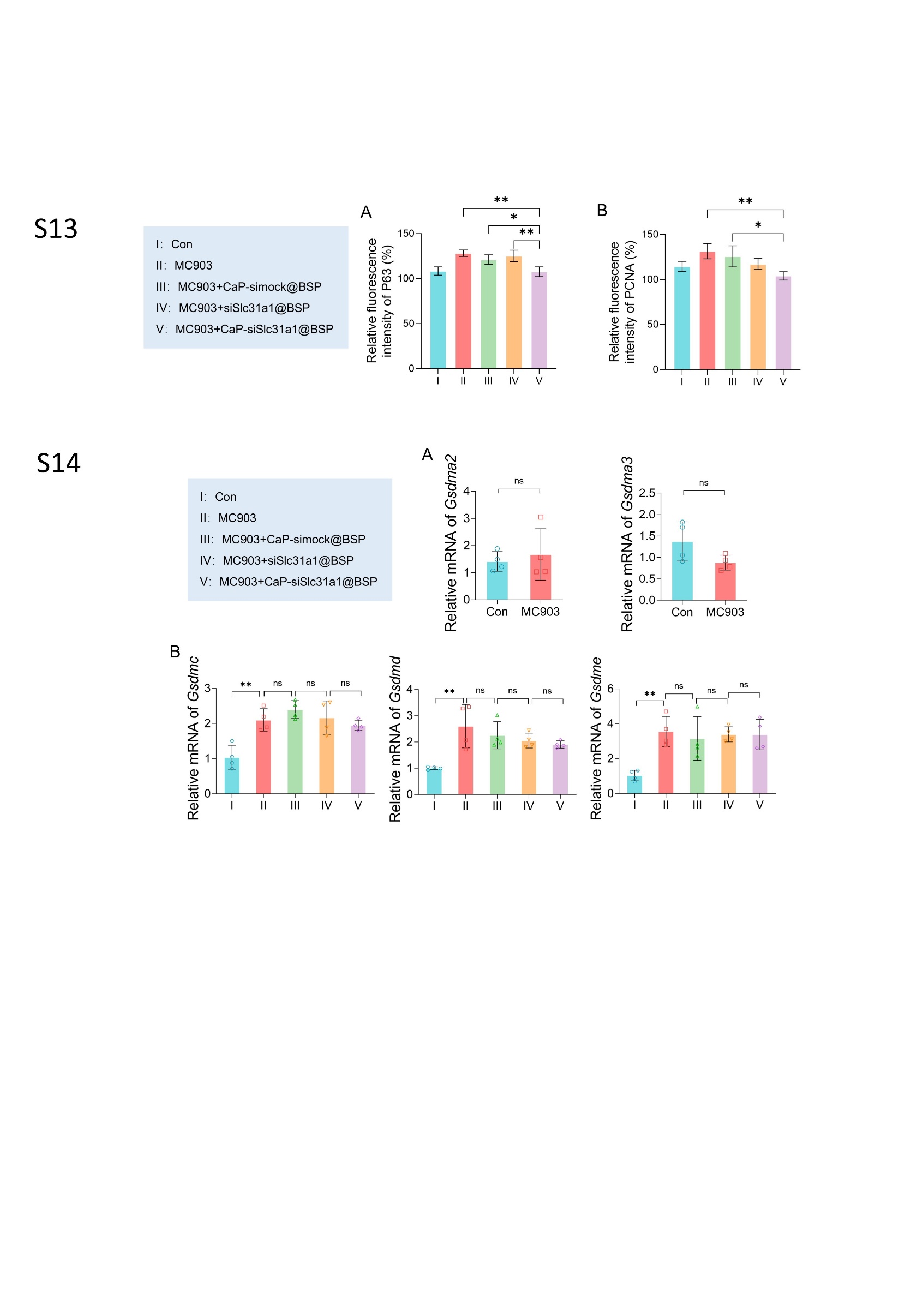


**Figure S13.** Statistical data on the expression of P63 and PCNA in skin lesions of mice following specified treatments.


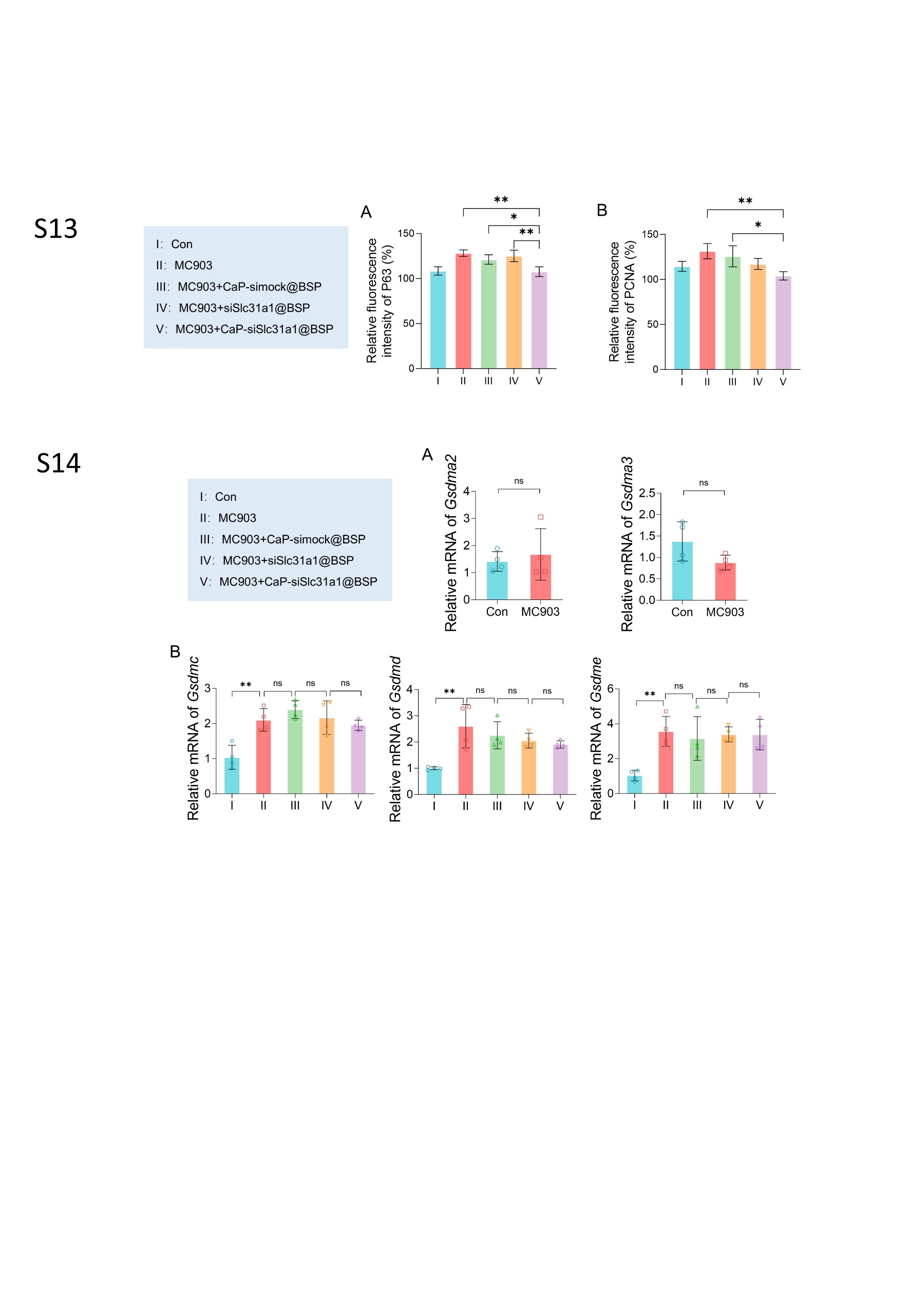


**Figure S14.** (A) Relative mRNA levels of *Gsdma2* and *Gsdma3* in skin lesions of mice following specified treatments (n = 4). (B) Relative mRNA levels of *Gsdmc*, *Gsdmd*, and *Gsdme* in skin lesions of mice following specified treatments (n = 4).

**Table S1. A list of primers used for qRT-PCR.**

| \| **Gene** \| **Species** \|  \| **Primer sequence** \| \| --- \| --- \| --- \| --- \| \| Cxcl1 \| mice \| Forward \| GCAGACCATGGCTGGGATTC \| \|  \|  \| Reverse \| AAGCCTCGCGACCATTCTTG \| \| Cxcl2 \| mice \| Forward \| AAAATCATCCAAAAGATACTGAACAA \| \|  \|  \| Reverse \| CTTTGGTTCTTCCGTTGAGG \| \| Cxcl3 \| mice \| Forward \| CCACCAACCACCAGGCTACAG \| \|  \|  \| Reverse \| GGCAAACTTCTTGACCATCCTTGAG \| \| Il4 \| mice \| Forward \| AGTTGTCATCCTGCTCTTCTTTCTC \| \|  \|  \| Reverse \| ATGGCGTCCCTTCTCCTGTG \| \| Il1b \| mice \| Forward \| GCAACTGTTCCTGAACTCAACT \| \|  \|  \| Reverse \| ATCTTTTGGGGTCCGTCAACT \| \| Il6 \| mice \| Forward \| GAGAGGAGACTTCACAGAGGATACC \| \|  \|  \| Reverse \| TCATTTCCACGATTTCCCAGAGAAC \| \| Il13 \| mice \| Forward \| CTTGCTTGCCTTGGTGGTCTC \| \|  \|  \| Reverse \| GGAGTCTGGTCTTGTGTGATGTTG \| \| Il15 \| mice \| Forward \| ACGTGCTCTACCTTGCAAACAGC \| \|  \|  \| Reverse \| TTCTCCTCCAGCTCCTCACATTCC \| \| Il31 \| mice \| Forward \| ACACAGGAACAACGAAGCCTACC \| \|  \|  \| Reverse \| ATATTGGGGCACCGAAGGACAAG \| \| Tslp \| mice \| Forward \| GTACGGATGGGGCTAACTTACAAC \| \|  \|  \| Reverse \| TGGCTTGCTCTCACAGTCCTC \| \| Slc31a1 \| mice \| Forward \| GGGGATGAGCTATATGGACTCC \| \|  \|  \| Reverse \| TCACCAAACCGGAAAACAGTAG \| \| Tnf \| mice \| Forward \| CACGCTCTTCTGTCTACTGAACTTC \| \|  \|  \| Reverse \| CTTGGTGGTTTGTGAGTGTGAGG \| \| Ifng \| mice \| Forward \| AACTCAAGTGGCATAGATGTGGAAG \| \|  \|  \| Reverse \| TCAAAGAGTCTGAGGTAGAAAGAGATAATC \| \| Gsdma1 \| mice \| Forward \| TCCCTTCTGCCTGGTGCTGAG \| \|  \|  \| Reverse \| GTTGCCACTGTCTGTCGGATCTG \| \| Gsdma2 \| mice \| Forward \| AGTGACCCTTGAAGCACTCC \| \|  \|  \| Reverse \| CGCAGGGGGAAAACATCCT \| \| Gsdma3 \| mice \| Forward \| CAGCTTGGCTCTACTGGGAC \| \|  \|  \| Reverse \| CGCCTGATCTTAGGGAAGGTTT \| \| Gsdmc \| mice \| Forward \| CCTTTCCAATGAGATTTGTGGG \| \|  \|  \| Reverse \| GGAAACTGGAGAACACAACTTC \| \| Gsdmd \| mice \| Forward \| GTGGACAGCCTGCGGAACTC \| \|  \|  \| Reverse \| GGTTCTGGTTCTGGAGCACTGG \| \| Gsdme \| mice \| Forward \| TGCTTGCTCCATGCTCTGTCTG \| \|  \|  \| Reverse \| ATGCCAAACCTCTCTGTGTCTCTC \| \| Fdx1 \| mice \| Forward \| CTAACGACCAAGGGGAAAATTG \| \|  \|  \| Reverse \| GGTCAAGCATGTCATTCTCTTC \| \| Dlat \| mice \| Forward \| ACCTCAGGCATGTATTCTGGCAATC \| \|  \|  \| Reverse \| CTCCAACTGCTCCATCCACAACTC \| \| Lias \| mice \| Forward \| TTTTGACCAGTCTCTTCGTGTA \| \|  \|  \| Reverse \| CTTTCAGTGTGGCATAGACTTG \| \| Atp7a \| mice \| Forward \| GAGGAGGCACAGACATCAA \| \|  \|  \| Reverse \| CCACACCAAGAGGGTAACA \| \| Atp7b \| mice \| Forward \| GGAAAGGCAGAGGTCAAGT \| \|  \|  \| Reverse \| ACAGGAAGCACAGGTCATC \| \| β-actin \| mice \| Forward \| GCTCTGGCTCCTAGCACCAT \| \|  \| Reverse \| GCCACCGATCCACACAGAGT \|   **Table S2. A list of primers for used ChIP.** | | | |
| --- | --- | --- | --- | --- | --- | --- | --- | --- | --- | --- | --- | --- | --- | --- | --- | --- | --- | --- | --- | --- | --- | --- | --- | --- | --- | --- | --- | --- | --- | --- | --- | --- | --- | --- | --- | --- | --- | --- | --- | --- | --- | --- | --- | --- | --- | --- | --- | --- | --- | --- | --- | --- | --- | --- | --- | --- | --- | --- | --- | --- | --- | --- | --- | --- | --- | --- | --- | --- | --- | --- | --- | --- | --- | --- | --- | --- | --- | --- | --- | --- | --- | --- | --- | --- | --- | --- | --- | --- | --- | --- | --- | --- | --- | --- | --- | --- | --- | --- | --- | --- | --- | --- | --- | --- | --- | --- | --- | --- | --- | --- | --- | --- | --- | --- | --- | --- | --- | --- | --- | --- | --- | --- | --- | --- | --- | --- | --- | --- | --- | --- | --- | --- | --- | --- | --- | --- | --- | --- | --- | --- | --- | --- | --- | --- | --- | --- | --- | --- | --- | --- | --- | --- | --- | --- | --- | --- | --- | --- | --- | --- | --- | --- | --- | --- | --- | --- | --- | --- | --- | --- | --- | --- | --- | --- | --- | --- | --- | --- | --- | --- | --- | --- | --- | --- | --- | --- | --- | --- | --- | --- | --- | --- | --- | --- | --- | --- | --- | --- | --- | --- | --- | --- | --- | --- | --- | --- |
| **Gene** | **Product**  **Length** |  | **Primer sequence** |
| Gsdma Primer1  (-1100bp, -800bp) | 300 | Forward | TGCTCTGTCGCCCAGCCT |
|  |  | Reverse | GGCTTGAGTGTGTGGGAA |
| Gsdma Primer2  (-1400bp, -1100bp) | 300 | Forward | CCCACCTCTGCCTCTCCT |
|  |  | Reverse | TGTCCCCAAAGTCTCATT |
| Gsdma Primer3  (-1700bp, -1400bp) | 300 | Forward | CAAGTGGAGTAATGACTA |
|  |  | Reverse | TCAAACAGTTCTCTGCCT |
| Gsdma Primer4  (-2000bp, -1700bp) | 300 | Forward | ACCCGGGAGGTGGAAGTT |
|  |  | Reverse | TCAAATGTTGCCTACATG |
| **Table S3. A list of primers for used ChIP.** | | | |
| **Gene** | **Product**  **Length** |  | **Primer sequence** |
| Gsdma Primer1  (-300bp, -1bp) | 300 | Forward | GCGAAACCCCGTCTCTAA |
|  |  | Reverse | GTATAACCTGACACAGCA |
| Gsdma Primer2  (-600bp, -300bp) | 300 | Forward | CTGCACATATGCATGGCC |
|  |  | Reverse | GTTTGTGTTGTTTAGTGT |
| Gsdma Primer3  (-900bp, -300bp) | 300 | Forward | CCATTTGGTCTAACTCCA |
|  |  | Reverse | GTGTGTGTGTGTGTGTGT |
| Gsdma Primer4  (-1200bp, -900bp) | 300 | Forward | ACTCCCACTGCCTCCCTC |
|  |  | Reverse | GACCATCCCTTGCCAGGA |
