# Supplementary material for: Microneedle-mediated transdermal delivery of siRNA-loaded nanoparticles for atopic dermatitis therapy by disrupting cuproptosis-pyroptosis crosstalk
Source: J Nanobiotechnology. 2026 May 18;24:654. doi: 10.1186/s12951-026-04533-9 (PMC13362213; doi:10.1186/s12951-026-04533-9)

Figure 1E

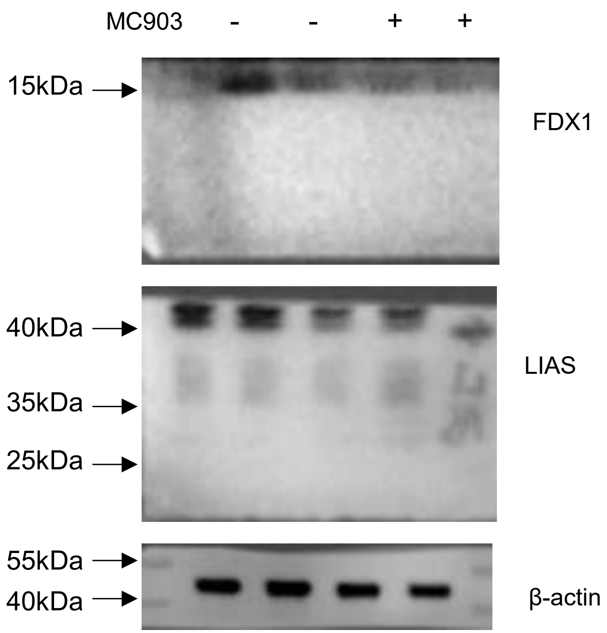

Figure 1F

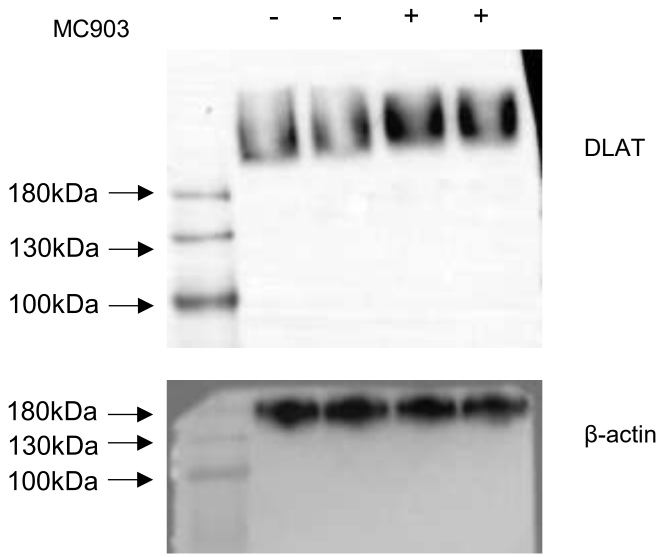

Native-PAGE Gel

Figure 3F

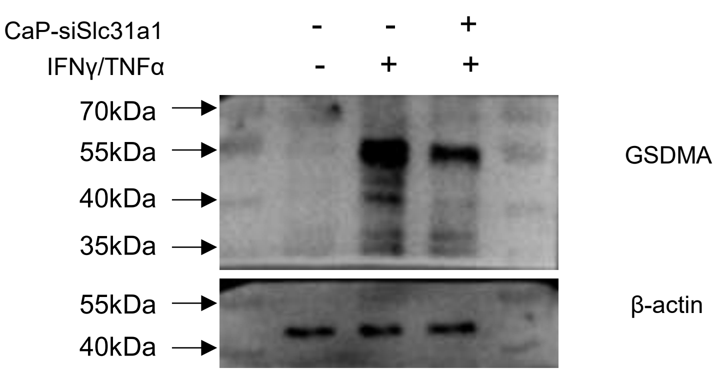

Figure 3G

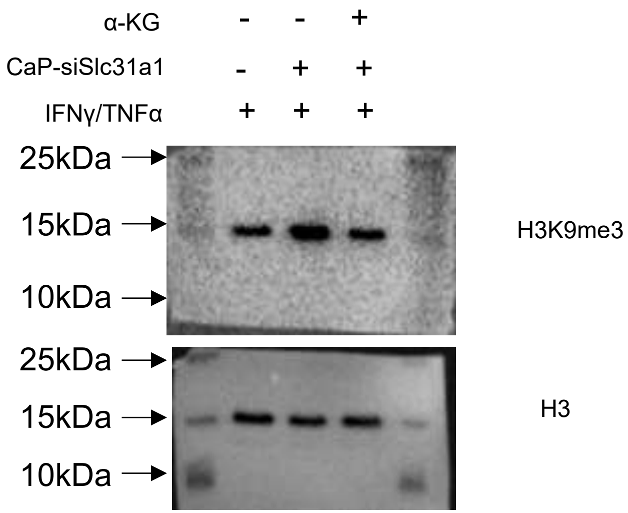

Figure 5E

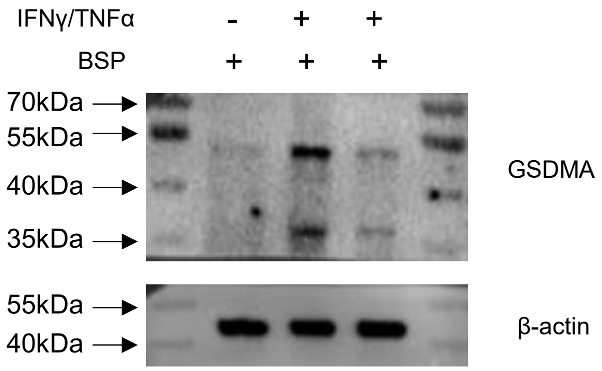

Figure 5F

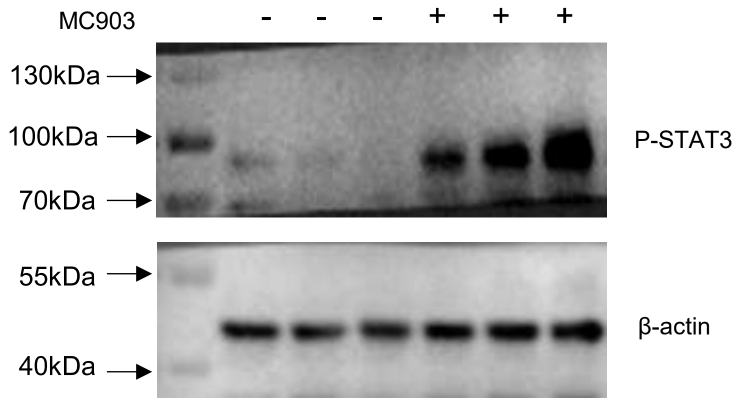

Figure 5G

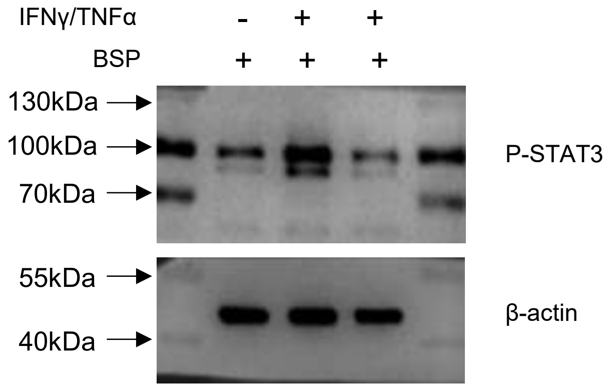

Figure 7I

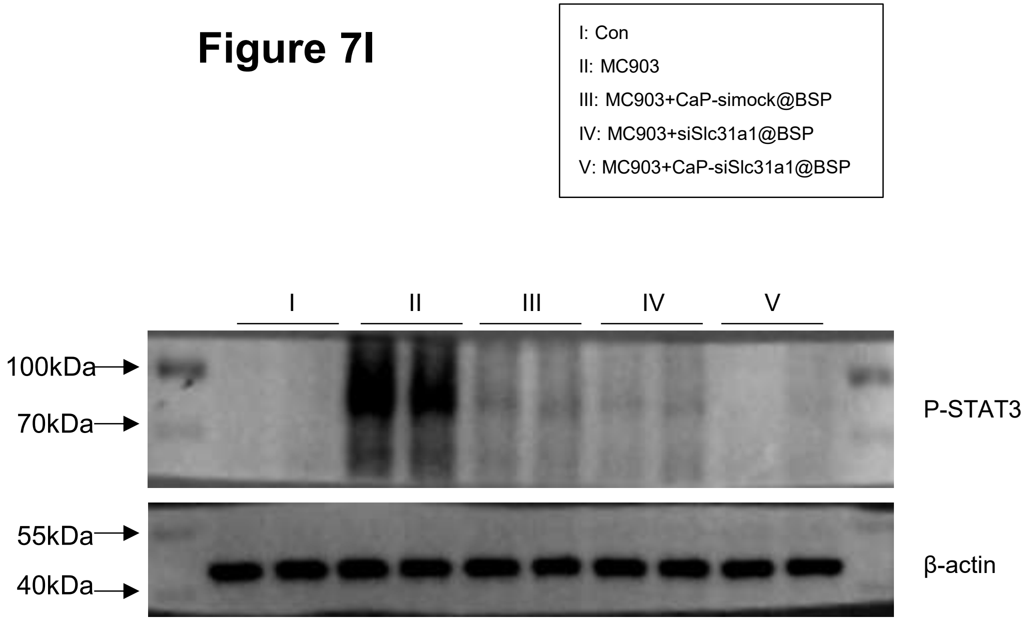

Figure 7J

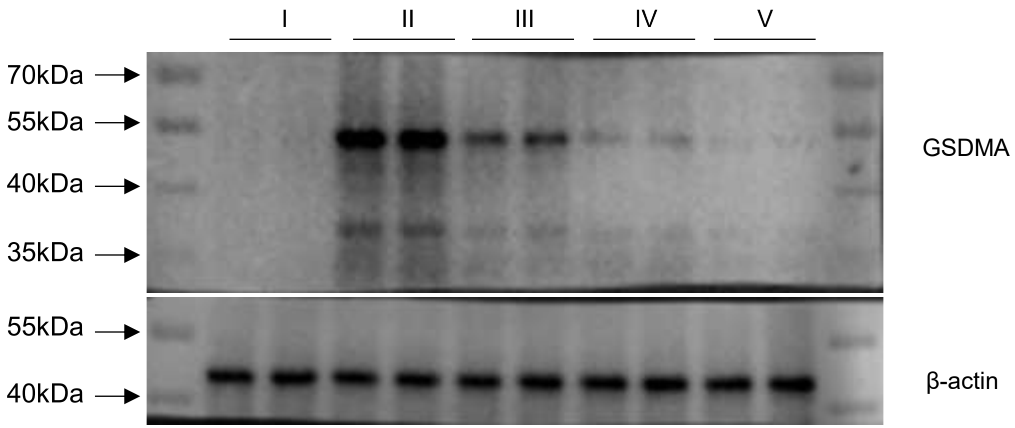

Supplement: Supplementary file 2 — Additional file 2. [file 12951_2026_4533_MOESM2_ESM.pdf]
